# Periodic formation of epithelial somites from human pluripotent stem cells

**DOI:** 10.1038/s41467-022-29967-1

**Published:** 2022-04-28

**Authors:** Marina Sanaki-Matsumiya, Mitsuhiro Matsuda, Nicola Gritti, Fumio Nakaki, James Sharpe, Vikas Trivedi, Miki Ebisuya

**Affiliations:** 1 European Molecular Biology Laboratory (EMBL) Barcelona, Barcelona, Spain; 2 EMBL Heidelberg, Developmental Biology Unit, Heidelberg, Germany

**Keywords:** Morphogenesis, Stem cells, Pattern formation, Stem-cell differentiation, Cell signalling

## Abstract

During embryonic development, epithelial cell blocks called somites are periodically formed according to the segmentation clock, becoming the foundation for the segmental pattern of the vertebral column. The process of somitogenesis has recently been recapitulated with murine and human pluripotent stem cells. However, an in vitro model for human somitogenesis coupled with the segmentation clock and epithelialization is still missing. Here, we report the generation of human somitoids, organoids that periodically form pairs of epithelial somite-like structures. Somitoids display clear oscillations of the segmentation clock that coincide with the segmentation of the presomitic mesoderm. The resulting somites show anterior-posterior and apical-basal polarities. Matrigel is essential for epithelialization but dispensable for the differentiation into somite cells. The size of somites is rather constant, irrespective of the initial cell number. The amount of WNT signaling instructs the proportion of mesodermal lineages in somitoids. Somitoids provide a novel platform to study human somitogenesis.

## Abstract (web summary)

Somitogenesis has been well characterized in model organisms, resulting in detailed description of the somite segmentation clock. Here they generate somitogenic organoids from human pluripotent stem cells that recapitulate somitogenesis, periodic segmentation, and proper polarity.

## Introduction

Somites are transient blocks of cells that give rise to a variety of tissues, including the vertebrae, rib cage, skeletal muscle, and part of the skin, in vertebrate embryos^1–3^. Bilateral pairs of somites periodically bud off from the presomitic mesoderm (PSM) along the anterior-posterior axis: as the mesenchymal cells migrate from the posterior PSM region near the tailbud to the anterior PSM region, they undergo a mesenchymal-epithelial transition (MET), acquiring the apical-basal polarity and elongated shapes. The anterior PSM cells eventually form spherical epithelial somites surrounding a core of mesenchymal cells. The somite formation, or somitogenesis, starts around day 20 after fertilization (Carnegie stage 9) in human embryos, and a total of ~40 pairs of somites are formed^4^. The timing of sequential somitogenesis is controlled by the segmentation clock, a molecular oscillator that peaks its activity every 5-6 hours in humans^2,5–9^.

Several aspects of somitogenesis have recently been recapitulated in vitro with pluripotent stem cells. PSM-like flat tissues made from mouse embryonic stem cells (ESCs) display the segmentation clock accompanied by tissue boundary formation^10^. Gastruloids are ESC-derived embryonic organoids that mimic early developmental events, including three-germ layer differentiation and axis patterning^11–13^, and mouse gastruloids embedded in an extracellular matrix (ECM) surrogate, Matrigel, form a string of single somites^14^. Mouse ESC-derived trunk-like structures (TLSs) form both the neural tube and bilateral somites^15^. Regarding human somitogenesis, several groups have induced PSM and somite cell fates from human pluripotent stem cells in 2D cultures and recapitulated the segmentation clock^6–9,16–18^. However, somites or epithelial structures are not formed in those human stem cell-derived models. Although 3D human ‘somitoids’ have recently been reported, the somite-like structures are not sequentially formed^19^. Thus, there is no in vitro model of human somitogenesis so far that recapitulates both the periodic somite formation coupled with the segmentation clock and the maturation into epithelial structures.

In this study, we report human embryonic organoids that periodically form pairs of somite-like structures with the mature epithelial organization along the anterior-posterior axis.

## Results

### Human iPSCs self-organize into somite-like structures

**Fig. 1:**
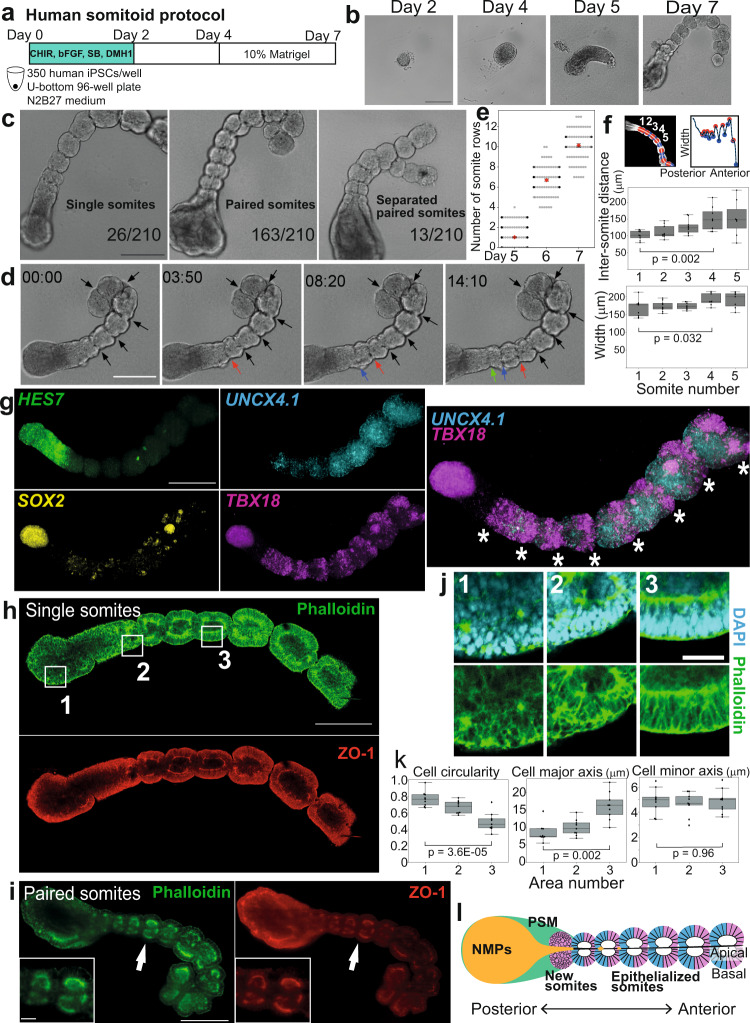
Generation and characterization of human somitoids. **a** Somitoid protocol. 350 human iPSCs were aggregated in a U-bottom plate and treated with CHIR99021, bFGF, SB431542, and DMH1. The cocktail was washed out on day 2, and Matrigel was added on day 4. **b** Time-course images of somitoid development. **c** Classification of morphologies of day 7 somitoids. *N* = 210 from 12 independent experiments. Only the images with entire somitoid structures were used. **d** Somite formation on day 6. Arrows indicate the somite boundaries. **e** Quantification of the number of somite rows. Mean ± SEM. *N* = 284 (day 5), 113 (day 6), and 77 (day 7). 3–15 independent experiments. **f** The images of day 7 somitoids were segmented, and the width perpendicular to the detected posterior-anterior axis was plotted (top). The somite-to-somite distance and the somite width (middle and bottom). Only somitoids with single somites were measured. Boxplots show median, 75th and 25th percentiles, and max and min. *N* = 9 (somite number 1–3), 8 (somite number 4), and 7 (somite number 5). P-values are from two-sided paired t-test. **g** HCR images of day 6 somitoids. Asterisks indicate the stripes of *TBX18* expression. *N* = 8 samples showed similar expression patterns. **h** IHC images of day 6 somitoid that displayed mostly single somites. *N* = 10 samples showed similar expression patterns. **i** IHC images of day 7 somitoids that displayed typical paired somites. *N* = 5 samples showed similar expression patterns. Insets: Enlarged images of the regions indicated by the arrows. **j** Enlarged images of the boxed regions in **h**. **k** Cell morphometry of Phalloidin images in j. Boxplots show median, 75th and 25th percentiles, and max and min except for outliers. *N* of measured cells = 9 (Area 1), 9 (Area 2), and 9 (Area 3). *P*-values are from two-sided student’s t-test. **l** Schematic diagram of a human somitoid. NMPs: Neuromesodermal progenitors, PSM: Presomitic mesoderm. Scale bars: 300 µm (**b**, **c**, **d**, **g**, **h**, **i**) and 50 µm (inset of **i**, **j**). Microscopes: Opera (**b**, **c**, **d**) FV3000 confocal (**h**, **j**), and Light-sheet (**g**, **i**). Source data are provided as a Source Data file.

To make human organoids that form somite-like structures (hereafter referred to as somitoids), we first made aggregates of human induced pluripotent stem cells (iPSCs) in a low attachment U-bottom plate (Fig. 1a). The aggregates were treated for 2 days with a cocktail of signaling molecules and inhibitors that has been used previously to induce the human PSM cell fate in 2D culture conditions^7,8,17^. The cocktail comprises CHIR99021 (a WNT signaling activator through the inhibition of GSK3β), bFGF, SB431542 (a TGFβ signaling inhibitor), and DMH1 (a BMP signaling inhibitor), mimicking WNT and FGF activation as well as TGFβ and BMP inhibition seen in the presumptive PSM region of mouse and chick embryos^17^. After 2-day treatment, the cocktail was gradually diluted by medium changes. The aggregates became oval-shaped around days 3-4 and then further elongated, reminiscent of gastruloids and TLSs^11–15^ (Fig. 1b; Supplementary Fig. 1a). Matrigel was added at a 10% concentration on day 4, and the somite-like ball structures first appeared around days 4-5 (Supplementary Fig. 1a). Approximately 10 (pairs of) somites were found per somitoid on day 7 (Fig. 1c–e).

Time-lapse imaging revealed that these somites were sequentially formed (Fig. 1d; Supplementary Movie 1). Either a single somite (12.4%) or a pair of somites (77.6%) was formed at one time, and occasionally (6.2%), the paired somites were completely separated (Fig. 1c; Supplementary Movie 1). Although most somitoids showed a single anterior-posterior axis, 3.8% of samples showed multiple axes (Supplementary Fig. 1b, c). Overall, the human somitoid protocol was reproducible: 210/210 samples, cultured over different batches, formed at least 3 (pairs of) somites by day 7 (Supplementary Fig. 1b).

The average length (along the anterior-posterior axis) and width of newly formed single somites were 116 µm and 157 µm, respectively, while those of paired somites, measured as individual somites, were 110 µm and 104 µm, respectively (Supplementary Fig. 2a). Namely, the somites formed as pairs were smaller than the single somites. Even though precisely measuring the size of somites of human embryos is challenging due to the limited resources, the somites of somitoids were in a comparable size range of in vivo human somites or in vitro mouse somite-like structures^15,20^ (Supplementary Fig. 2). The somite width and the somite-to-somite distance showed a gradual increase from the posterior (newly formed) somites to the anterior (older) somites (Fig. 1f), as reported in chicken embryos and mouse gastruloids^14,21^. The older somites also tended to be more spherical (Supplementary Fig. 3; see Fig. 1c, for example), possibly due to dominant tissue surface tension in the absence of constraints from neighboring tissues^15,22,23^.

### Somitoids possess proper polarities

To molecularly characterize somitoids, we examined lineage marker expressions with qRT-PCR analyses (Supplementary Fig. 4). The expression levels of pluripotency markers, such as *NANOG, OCT4/POU5F1*, and *SOX2*, dramatically declined by day 4. The *SOX2* expression recovered after the drop, probably because it is also expressed in neuromesodermal progenitors (NMPs)^24–26^. By contrast, a mesodermal marker *BRACHYURY* (also known as *TBXT*, expressed both in mesoderm and NMPs) showed an expression peak on day 4. PSM markers *TBX6* and *HES7*^27^ as well as a segmentation maker *MESP2*^28^ also peaked on days 4-5. Somite markers *UNCX4.1* (also known as *UNCX*)^1^, *TCF15*^16^, and *FOXC2*^16^, showed higher expressions after day 5.

To further examine the spatial gene expression patterns, we visualized the marker genes with immunostaining and in situ hybridization chain reaction (HCR)^29^. The co-expression of BRACHYURY (a mesodermal marker) and SOX2 (a neural marker) in somitoids indicates the existence of NMPs, bipotent progenitor cells that give rise to both mesodermal and neural lineages^24–26^ (Supplementary Fig. 5). The NMP region at the posterior end of somitoids was surrounded by PSM marker (TBX6 and *HES7*)-positive cells, and the PSM region extended slightly more anteriorly (Supplementary Fig. 5; Fig. 1g). By contrast, a somite marker *UNCX4.1* was expressed in somite-like structures in the anterior regions of somitoids (Fig. 1g). The boundary between *HES7* and *UNCX4.1* corresponds to the newest somite formed from PSM. *TBX18*, a marker for the rostral halves of somites^1^, showed stripe patterns in somites as reported in the somite-like structures of mouse gastruloids^14^. The alternating patterns of *UNCX4.1* (a marker for the caudal halves of somites) and *TBX18* indicate the rostral-caudal patterning in individual somites^1^ (Fig. 1g, right; Supplementary Fig. 6; Supplementary Movie 2), even though the patterns in somitoids are less clear than the in vivo counterparts^30^. These results demonstrate that the NMPs, PSM, and somites are properly located along the posterior-anterior axis of somitoids and that each somite has rostral and caudal compartments.

To examine the maturity of somites, we stained markers for epithelialization^31^. A tight junction marker ZO-1 showed clear localization to the inner surface of somites (Fig. 1h, i), demonstrating the establishment of the apical-basal polarity in somite cells and the formation of the apical lumen in somites. The F-actin staining with Phalloidin also showed a stronger signal on the apical surface of somites (Fig. 1h, i) and demarcated the elongated shapes of epithelial somite cells (Fig. 1j, k, Area 3). The nuclei aligned along the basal surface of mature somites (Fig. 1j, Area 3). By contrast, the F-actin and nuclear stainings showed more random patterns in the PSM/NMP regions, and those immature mesenchymal cells had rounded morphologies (Fig. 1j, k, Area 1). These results demonstrate that a MET happens while PSM cells differentiate into somite cells along the posterior-anterior axis of somitoids and that the formed epithelial somites are mature enough to establish the apical-basal polarity.

Between paired epithelial somites, we sometimes observed a line of cells (Supplementary Fig. 7a). However, those cells did not display any continuous structure that resembles a neural tube or notochord. Some cells at the center were BRACHYURY and SOX2 double-positive (Supplementary Fig. 7b; Supplementary Movie 3), suggesting that they are NMPs. Thus, we concluded that somitoids display transcriptional and morphological hallmarks of somitogenesis but not those of neural tube or notochord formation and that the formed somites possess organized epithelial structures (Fig. 1l). This conclusion supports the idea that human somitogenesis is largely a tissue-autonomous process that does not require adjacent tissues, including the structured neural tube and notochord, consistent with previous results of mouse explant cultures and gastruloids^14,32^.

### Somitoids contain NMP, PSM, and somite cell populations

**Fig. 2:**
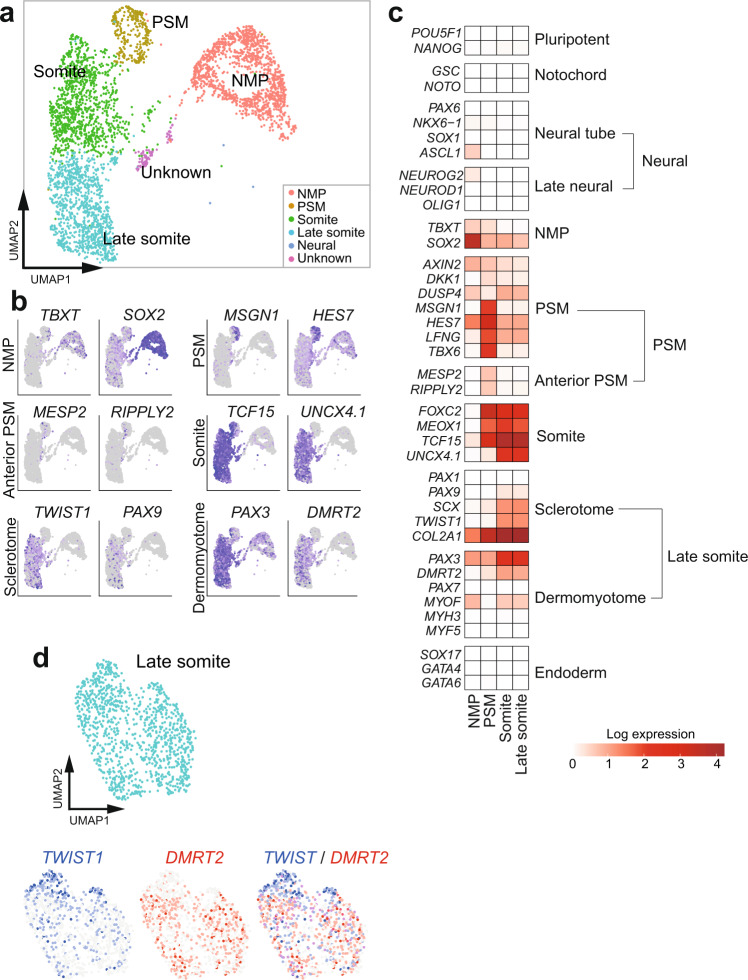
scRNA-seq analyses of somitoids. **a** Uniform manifold approximation and projection (UMAP) plot of day 7 somitoids with 10% Matrigel (*N* = 27 pooled samples, 3781 cells) colored by the 6 clusters identified in Supplementary Fig. 8a. NMP: Neuromesodermal progenitor, PSM: presomitic mesoderm. The cluster label for Neural is not shown as the cells in this cluster represent less than 0.1% of the total. **b** UMAP plots colored by the expression of selected marker genes. **c** Heatmap of the natural log expression of selected marker genes associated with early embryonic development. **d** Subset analysis of Late somite cells colored by the expression of sclerotome (*TWIST1*) and dermomyotome (*DMRT2*) markers (bottom).

To systematically characterize the cell types consisting of somitoids, we performed single-cell RNA sequencing (scRNA-seq) for day 7 somitoids (Fig. 2; Supplementary Figs. 8–9). Clustering analyses detected populations corresponding to putative NMP, PSM, somite, and late somite cells (Fig. 2a, b; Supplementary Fig. 8d, Sample1). The RNA velocity suggested the differentiation of somite cells from PSM cells (Supplementary Fig. 9a). By contrast, the differentiation of PSM cells from NMPs was not clear (Supplementary Fig. 9a), possibly because most PSM differentiation might happen in the early stages of somitoids in the presence of the cocktail of signaling molecules and inhibitors. Consistent with the lack of morphological features of a neural tube or notochord in somitoids, a clear neural, notochord-, or endoderm-like cell population was not detected (Fig. 2a, c; Supplementary Fig. 9b). In living embryos, the ventral and dorsal portions of somites further become the sclerotome and dermomyotome, respectively^1,3^. A couple of sclerotome markers, including *TWIST1* and *PAX9*, and dermomyotome markers, including *DMRT2* and *PAX3*, were expressed in the late somite cell population of somitoids^15^ (Fig. 2c, d; Supplementary Fig. 9e). The sclerotome and dermomyotome markers tended to show mutually exclusive patterns (Fig. 2d). These results suggest that somites in somitoids may have started differentiating into sclerotome and dermomyotome cells, even though somitoids do not show morphological features of sclerotomes and dermomyotomes.

We further characterized the developmental stage and cell proliferation patterns of somitoids by using expression patterns of *HOX* gene clusters and *PCNA*, respectively. The PSM, somite, and late somite cells in somitoids showed the continuous expression of *HOX* genes until *HOX9-10*, suggesting that somitoids mostly recapitulate thoracic and lumbar somitogenesis (Supplementary Fig. 9d). The cell proliferation marker *PCNA* was expressed in most regions of somitoids, but the expression was weaker in late somite cells (Supplementary Fig. 9c). Consistent with this, EdU incorporation into somitoids demonstrated that cells still proliferate in the regions of NMP, PSM, and the first 1-2 somites, while cell proliferation is not active in older somites (Supplementary Fig. 10).

### Somite formation is coupled with the segmentation clock

**Fig. 3:**
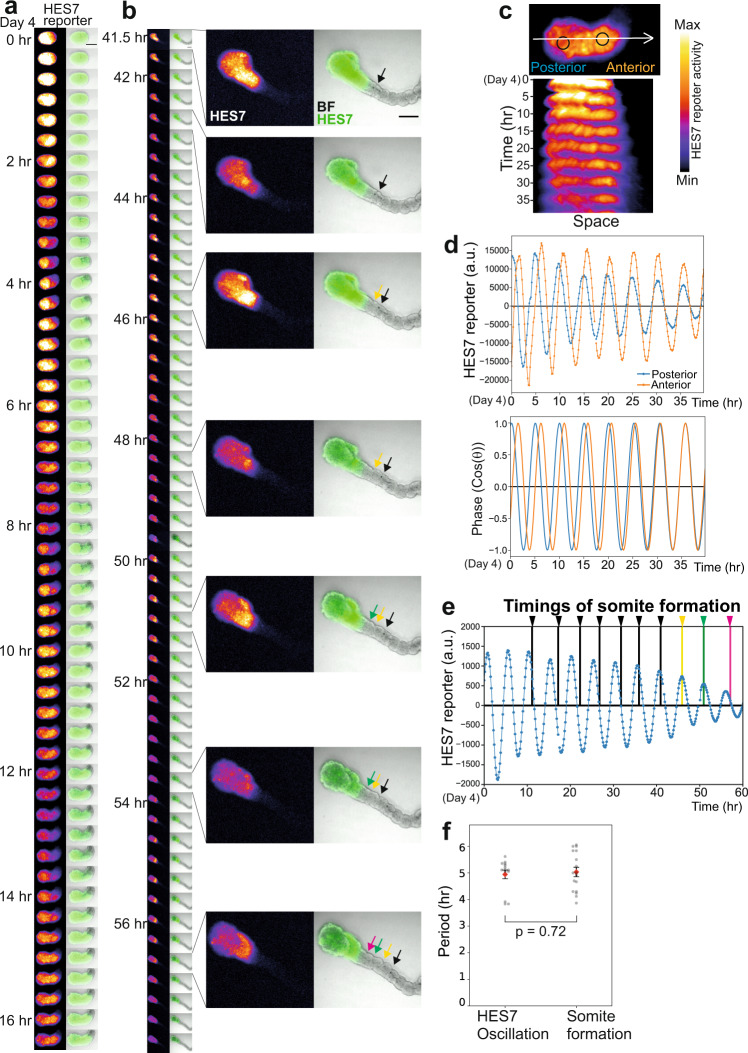
Somites are formed according to the segmentation clock. **a, b** Time-lapse images of the HES7 promoter-luciferase reporter activity in a representative somitoid. The starting point on day 4 was defined as time 0. See also Supplementary Movie 4. Scale bars: 500 µm. Images were taken by an LV200 luminescent microscope. **b** Enlarged images of several time points are also shown. Arrows indicate the somite boundaries. BF: Bright field. **c** Kymograph of the HES7 reporter activity measured along the posterior-anterior axis (the white arrow) of the same sample shown in **a**, **b**. **d** Detrended intensity (top) and the oscillation phase (bottom) of the HES7 reporter activity in the same sample shown in **a**, **b**. Blue and orange lines indicate the signals measured in the posterior and anterior regions of somitoids, respectively, marked in **c**. The gaps in the graph correspond to short halts of imaging to adjust the sample position. *N* = 14 samples showed similar oscillatory patterns. **e** Relationship between HES7 oscillations and segmentation timings. Detrended HES7 reporter activity of the entire image of the same sample shown in a and b. Arrows indicate the timings of somite formation, and the colors correspond to the arrows in **b**. **f** Periods calculated from the HES7 oscillations and somite formation. Mean ± SEM. Each point in the graph indicates an average period of several oscillation peaks or somite formation timings in one sample from day 5 to day 6. *N* = 14 (HES7 oscillation) and 17 (Somite formation). 5 independent experiments. *P*-value is from two-sided student’s t-test. Source data are provided as a Source Data file.

Multiple somite-like structures can be induced even in the absence of the segmentation clock when signaling pathways are modulated^33,34^. To test whether the somitogenesis in somitoids is regulated by the segmentation clock, we monitored the expression patterns of *HES7*, a core gene of the molecular oscillator^27,35^. The HES7 promoter-luciferase reporter^7,8^ exhibited clear collective oscillations immediately after the medium change on day 2 (Supplementary Fig. 11a). Postponing the medium change postponed the onset of collective oscillations, suggesting that the medium change entrains the oscillation phases of individual cells (Supplementary Fig. 11a). The HES7 reporter also showed traveling waves from the posterior PSM to the anterior PSM of somitoids (Fig. 3a, b; Supplementary Movie 4). The HES7 waves were easier to be detected on day 4 when somitogenesis was about to start (Fig. 3a, c): the HES7 oscillations in the posterior PSM preceded those in the anterior PSM, demonstrating traveling waves (Fig. 3c, d; Supplementary Fig. 11b). The oscillations and waves were disrupted by treatment with a NOTCH signaling inhibitor DAPT (Supplementary Fig. 12), consistent with the in vivo segmentation clock^2,3,36^.

On day 6, when somitogenesis was actively happening, the waves became less visible, but the oscillations were still clear (Fig. 3b, e). Importantly, the timings of HES7 oscillations coincided with those of somite formation: one somite or one pair of somites was produced during one oscillation cycle (Fig. 3e). The period of HES7 oscillations was ~5 hours (Fig. 3f), a typical period of the human segmentation clock^2,5–9^. The period calculated from the somite formation timings was also ~5 hours (Fig. 3f). These results indicate that the somite-like structures in somitoids are periodically formed according to the human segmentation clock.

### Matrigel is crucial for somite formation

We next explored the conditions to generate successful somitoids. Matrigel is a mixture of ECM components, and Matrigel embedding has been demonstrated to be crucial for somite formation in mouse stem cell-derived models^14,15^. The first somites were formed in somitoids ~10 hours after Matrigel addition on day 4 (Supplementary Fig. 13a, b). When Matrigel was added 4 hours later than the usual timing, the first somites were formed ~4 hours later than usual, suggesting that Matrigel addition is the trigger of the first somite formation (Supplementary Fig. 13a, b). In both conditions, the first somites were formed at the ~20% position of somitoids along their midline (Supplementary Fig. 13c, d).

**Fig. 4:**
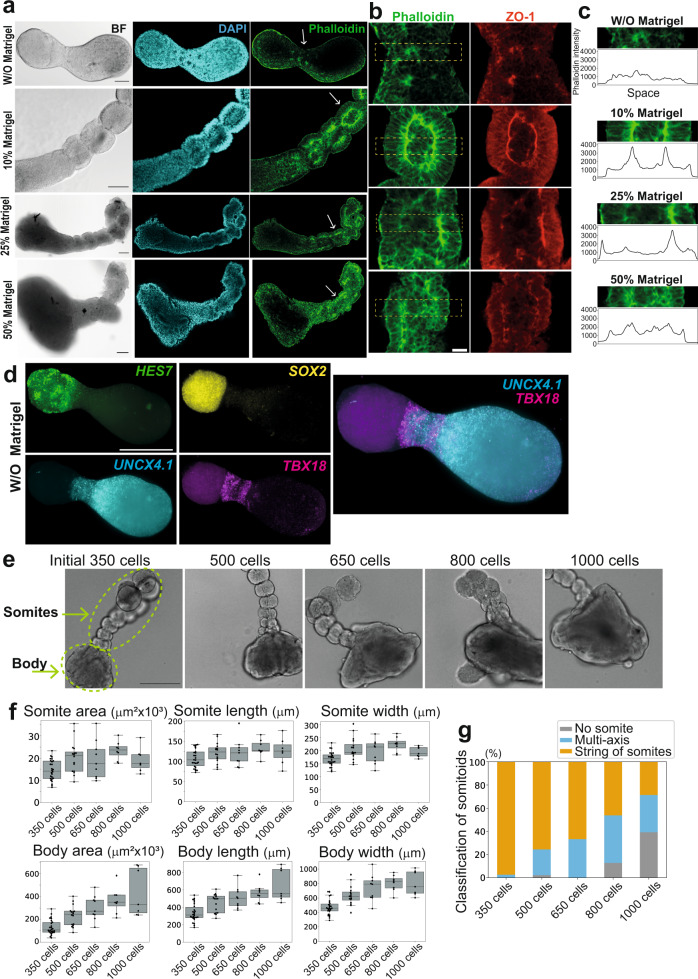
Effects of Matrigel and the initial cell number on somitoid morphologies. **a** Bright field (BF), DAPI staining, and Phalloidin staining images of the somitoids created without (W/O) or with different concentrations of Matrigel. Matrigel was added on day 4, and the images were taken on day 6. Scale bars: 100 µm. All samples stained (*N* = 6 (W/O Matrigel), 10 (10% Matrigel), 8 (25% Matrigel), and 5 (50% Matrigel)) showed similar expression patterns. **b** Enlarged images of the regions indicated by the white arrows in a. Different Z-planes were used between a and b to show the images clearly. ZO-1 staining images are also shown. Scale bars: 30 µm. **c** Enlarged images of the boxed regions in b and their intensity profiles. **d** HCR images of the day 7 somitoids without Matrigel. Scale bar: 300 µm. N = 4 samples showed similar expression patterns. **e** Representative images of the day 7 somitoids created from 350, 500, 650, 800, and 1000 cells. The light green dashed lines indicate a string of somites and a body that includes the PSM and NMPs. Scale bar: 350 µm. **f** Size measurements of the somite and body parts of the somitoids created with different initial cell numbers. The length and width mean the distances along the anterior-posterior axis and its perpendicular axis, respectively. Only the newest somites were measured. Boxplots show median, 75th and 25th percentiles, and max and min except for outliers. *N* = 26 (350 cells), 16 (500 cells), 9 (650 cells), 8 (800 cells), and 7 (1000 cells). **g** Classification of the somitoids created with different initial cell numbers. *N* = 150 (350 cells), 45 (500 cells), 24 (650 cells), 39 (800 cells), and 28 (1000 cells). 5–12 independent experiments. Part of samples of 350 cells is common to Fig. 1c. Microscopes: FV3000 confocal (**a**), Light-sheet (**d**), and Opera (**e**). Source data are provided as a Source Data file.

In the absence of Matrigel, our human somitoid protocol (Fig. 1a) never gave rise to a somite-like structure (Supplementary Fig. 14, W/O Matrigel). Instead, hourglass-shaped bodies were formed without Matrigel (Fig. 4a; Supplementary Movie 5, W/O Matrigel), and apical markers ZO-1 and F-actin showed polarized localization in small clusters of cells, especially near the narrow neck of the hourglass (Fig. 4b). However, the cell clusters with apical-basal polarities were small and sporadic, and they did not form sequential epithelial organizations with a consistent apical-basal polarity (Fig. 4b, c).

Interestingly, somitoids without Matrigel still displayed a proper anterior-posterior axis, expressing a somite marker *UNCX4.1* on the opposite side to a PSM marker *HES7* (Fig. 4d). Another somite marker *TBX18* showed a localized expression in the neck region of the hourglass. The expression levels of a MET marker *N-CADHERIN*^37^ checked with qRT-PCR analysis were also comparable between the absence and presence of Matrigel (Supplementary Fig. 14b). Our scRNA-seq analyses further confirmed that all the major cell types identified in the control somitoids (10% Matrigel), putative NMP, PSM, somite, and late somite cells, existed in the somitoids without Matrigel (Supplementary Fig. 15). Even some sclerotome and dermomyotome markers were similarly expressed in the absence of Matrigel (Supplementary Fig. 15a, e). These results suggest that Matrigel is dispensable for the axis elongation and somite cell differentiation but necessary for the epithelialization and the establishment of a consistent apical-basal polarity. In embryos, the PSM and somites are surrounded by the surface ectoderm, and the ECM produced from the surface ectoderm, particularly fibronectin, is essential for somite formation^15,32,38,39^. Matrigel may act as a surrogate for the surface ectoderm that signals the apical-basal polarity to somite-forming cells. Even though fibronectin is not a major component of Matrigel^40^, *FIBRONECTIN* (*FN1*) was expressed in somitoids (Supplementary Fig. 9c), and thus Matrigel may trap FIBRONECTIN secreted by organoids^15^.

While a low concentration (5%) of Matrigel resulted in similar somitoids as the control 10% Matrigel, high concentrations (25% and 50%) of Matrigel led to abnormal morphologies (Fig. 4a; Supplementary Fig. 14a; Supplementary Movie 5). Although the cells were still epithelialized with high concentration Matrigel, the somite shapes were not spherical but skewed and convoluted (Fig. 4b, c), and occasionally, multiple small somites were arranged like a ‘bunch of grapes’^15,33^ instead of a string of somites (Supplementary Fig. 14a; Supplementary Movie 5, 50% Matrigel). The skewed somites could be due to the stiffness of high concentration Matrigel, and these results highlight the importance of a proper concentration (5–10%) of Matrigel^41^.

### The somites have a preferred size

As another crucial condition in many types of organoids is the initial cell number^11,42–44^, we made somitoids with various numbers of human iPSCs ranging from 200 to 1000 cells. The somitoids created from 200 cells, instead of the control 350 cells, stopped growing early (Supplementary Fig. 16), and thus they were excluded from the following analyses. Above 350 cells, the size of the posterior ‘body’ that includes NMP and PSM regions monotonously increased according to the initial cell number (Fig. 4e, f; see Body morphometry; Supplementary Fig. 17). Surprisingly, however, the size of somites did not increase accordingly, but it was rather constant (Fig. 4e, f; see Somite morphometry). This suggests that the somites have a preferred size, which might be determined by local cell-cell interactions^33^, the segmentation clock, or other mechanisms. Note that, however, the somitoids with large initial cell numbers tended to form multiple axes and sometimes failed to form somites (Fig. 4g), indicating an optimal initial number of 350–500 cells.

### WNT signaling instructs lineage specification

**Fig. 5:**
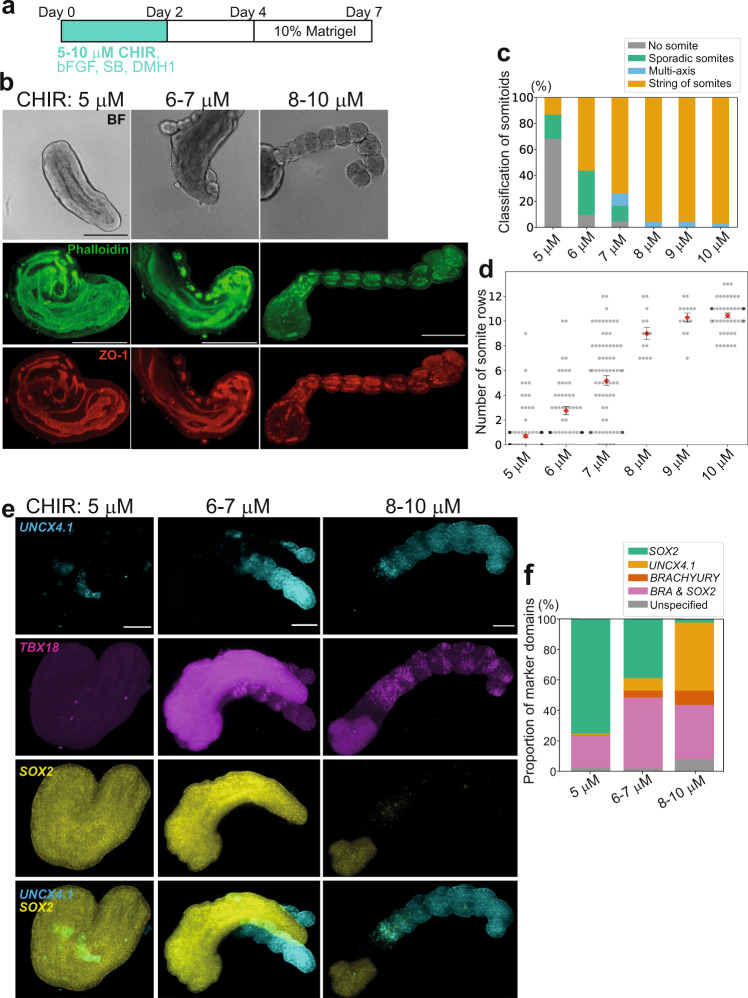
WNT signaling instructs the mesodermal lineage specification. **a** Somitoid protocol with altered CHIR concentrations during the initial 2 days. The original protocol uses 8–10 μM CHIR. **b** Bright field (BF), Phalloidin staining, and ZO-1 staining images of the day 7 somitoids created with different CHIR concentrations. BF samples are different from Phalloidin- and ZO-1-stained samples. Scale bars: 350 µm. All samples stained (*N* = 3 (5 µM), 7 (6-7 µM), and 5 (8–10 µM)) showed similar expression patterns. **c** Classification of the day 7 somitoids created with different CHIR concentrations. Sporadic somites mean only 1–2 isolated somites, whereas strings of somites mean more than 3 rows of somites. *N* = 113 (5 µM), 62 (6 µM), 126 (7 µM), 46 (8 µM), 45 (9 µM), and 150 (10 µM). 4–12 independent experiments. **d** Quantification of the number of somite rows in the day 7 somitoids created with different CHIR concentrations. Mean ± SEM. *N* = 112 (5 µM), 56 (6 µM), 77 (7 µM), 14 (8 µM), 14 (9 µM), and 49 (10 µM). 4–12 independent experiments. Only the images with the entire somitoid structures were measured. **c**, **d** Part of samples of 8–10 µM is common to Fig. 1c, e. **e** HCR images of day 6 somitoids. Scale bars: 150 µm. **f** Volume quantification of lineage marker expression domains using HCR images of day 6 somitoids. *SOX2* + & *BRACHYURY*-: Neural; *UNCX4.1* + : Somite; *BRACHYURY* + & *SOX2*-: PSM; *BRACHYURY* + *& SOX2* + : NMP. All samples stained (*N* = 3 (5 µM), 4 (6-7 µM), and 6 (8-10 µM)) showed similar patterns as long as somites were present. Microscopes: Opera (BF of panel **b**) and Light-sheet (Phalloidin and ZO-1 of **b**, **e**). Source data are provided as a Source Data file.

The amount of WNT signaling is known to instruct NMPs to become either the neural tube or PSM/somites:^15,25,26,45^ WNT activation promotes mesodermal lineage differentiation at the expense of neural lineages. Thus, we modulated the dosage of the WNT signaling activator CHIR during the initial 2 days of the somitoid protocol (Fig. 5a). High doses of CHIR (8-10 µM) of the original protocol resulted in usual somitoids with strings of somites (Fig. 5b; Supplementary Fig. 18a). Lowering the dosage decreased the possibility of somite formation (Fig. 5b–d; Supplementary Fig. 18). With 5 µM CHIR, most somite-like structures either disappeared or became sporadic. Instead, an elongated, large epithelial structure was observed (Fig. 5b; Supplementary Fig. 18a). The elongated epithelial structure was *SOX2*-positive (Fig. 5e, f), suggesting that it may be closer to the neural tube. With intermediate CHIR doses (6–7 µM), both the elongated epithelial structure and somites were formed (Fig. 5b, e; Supplementary Fig. 18a). The elongated epithelial structure was occasionally flanked by bilateral somites (Fig. 5e, 6–7 µM), reminiscent of the neural tube and bilateral somites formed in mouse TLSs^15^. The elongated epithelial structure was indeed positive for neural tube markers (SOX2, SOX1, and PAX6)^15^, whereas the somite-like structures were negative (Supplementary Fig. 19). Note that, however, long strings of somites were rarely observed with lower CHIR doses, and sporadic or short rows of somites were often formed (Fig. 5c, d; Supplementary Fig. 18b, c, 5–7 µM). The WNT sensitivity of somitoids was so high that even a 1 µM increase in the CHIR dosage dramatically changed the outcome (Supplementary Fig. 18a). These results suggest that somite formation in somitoids needs a high amount of WNT signaling to make mesodermal lineages, namely the PSM and somites, at the expense of neural lineages (Fig. 5f).

To confirm the effect of WNT signaling on the cell lineage specification in somitoids, we barcoded the somitoids that were created with 5 µM, 7 µM, and 10 µM CHIR and performed MULTI-seq analyses^46^ (Supplementary Figs. 20–22). As expected, the populations of putative NMP, PSM, somite, and late somite cells were identified as the major cell types in the somitoids with the high CHIR dose (Supplementary Fig. 20b, c, 10 µM). With the low CHIR dose, by contrast, neural tube markers *SOX1* and *PAX6* were expressed in the somitoids (Supplementary Fig. 21, 5 µM), and the putative neural cells and NMPs were the major cell types in addition to the minor somite cell population (Supplementary Fig. 20b, c, 5 µM). RNA velocity demonstrated the differentiation of the neural cells from NMPs (Supplementary Fig. 20e). The neural cell population was not detected in the somitoids with the high CHIR dose (Supplementary Fig. 20b, c, 10 µM). All these cell types, including both neural and mesodermal lineages, were detected in somitoids with the intermediate CHIR dose (Supplementary Fig. 20b, c, 7 µM). Gene ontology (GO) terms related to neural and mesodermal differentiation were enriched in the genes upregulated by 5 µM and 10 µM CHIR, respectively (Supplementary Fig. 20d; Supplementary Data 1). Thus, the MULTI-seq comparison clearly depicted the bias towards neural or mesodermal lineages depending on the low or high amount of WNT signaling, respectively, during the initial 2 days of the somitoid protocol (Supplementary Figs. 21–22). The day 3 somitoids created with 10 µM CHIR already expressed a PSM marker TBX6 while those with 5 µM CHIR did not (Supplementary Fig. 23), suggesting that the bias in the lineage specification in somitoids already starts on day 3 when somites are yet to be formed.

## Discussion

We created human somitoids that recapitulate periodic somite formation coupled with the oscillations of the segmentation clock. The somitoids elongated, and the anterior PSM cells rhythmically budded off to make pairs of somites. The resulting somite tissues were mature enough to display the caudal-rostral compartments and apical-basal polarity.

Although the somite cells in somitoids expressed a couple of sclerotome and dermomyotome markers, we did not notice morphological changes corresponding to the ventral sclerotomes and dorsal dermomyotomes of in vivo embryos. We reason that for further somite maturation, inductive signals coming from the neighboring tissues, such as the neural tube, notochord, and lateral plate mesoderm, should be essential^1^. Treating somitoids with additional growth factors and inhibitors may be a way to make more mature sclerotomes and dermomyotomes. As our somitoids displayed the neural tube-like structure flanked by somites with lower doses of the WNT activator, making several neighboring tissues altogether with somites may be an alternative way.

Like many other epithelial organoids, somitoids needed Matrigel to form somites. Matrigel is currently a ‘magic powder’ in the organoid field, and its precise role remains unclear. Since PSM cells differentiated into the somite fate even in the absence of Matrigel, the ECM surrogate may play a role mainly in cell epithelialization and apical-basal polarization. In addition, the fact that epithelialization itself happened with a wide range of Matrigel concentrations (5–50%) suggests that the accurate stiffness of the gel may not be important for epithelialization. Another unexpected finding regarding the somitoid protocol was that the size of somite was relatively constant irrespective of the initial cell number. This was surprising because the somite size has been proposed to scale with the size of the PSM region^47–49^. Further investigation of the effect of cell number on the segmentation clock, cell differentiation, cell movement, and tissue mechanics will be necessary to address the question^23,33,39,50,51^.

Even though the somitoid protocol was reproducible, it was sensitive to experimental materials and cell states. For instance, we found that the combination of a particular iPSC line, culture medium, and even U-bottom plate was crucial for somitoids. The morphology of iPSC colonies was also important. The incompatible materials and troubleshooting are summarized in a separate protocol^52^.

Human somitoids should provide a novel platform to study congenital abnormalities in vertebral segmentation, including spondylocostal dysostosis (SCD) and congenital scoliosis^5,53^. We previously modeled SCD phenotypes by recapitulating the human segmentation clock from patient-derived iPSCs^7^. Whereas the in vitro segmentation clock revealed congenital defects in oscillation patterns, the new somitoid model will enable us to study defects related to somite formation and epithelialization. As somitogenesis is sensitive to developmental environments, such as hypoxia^54^, somitoids may also be useful for high-throughput assessments of environmental factors and teratogens. Another interesting direction is the inter-species comparison of somitogenesis. We have previously compared the human and mouse segmentation clocks, demonstrating the oscillation period difference between the species^8,55^. The new human somitoid model will enable the comparative study on how the tissue dynamics and morphogenesis of somite formation are regulated across species^56^.

## Methods

### Human iPSC cultures

Human iPSC line (201B7 line, #HPS0063)^57^ was provided by the RIKEN BRC through the National BioResource Project of the MEXT/AMED, Japan. The human iPSCs were maintained without feeder cells and cultured on iMatrix-511 silk (Amsbio, 892021)-coated dishes in StemFit medium (Ajinomoto, StemFit Basic04CT). The cells were trypsinized into single cells by TrypLE solution (Gibco, A1285901) and 0.5 mM ethylenediaminetetraacetic acid (EDTA) in phosphate-buffered saline (PBS) (1:1 mixture). 1.2 × 10^4^−2.4 × 10^4^ cells were seeded into an iMatrix-coated 3.5 cm dish in the StemFit medium containing 10 µM ROCK inhibitor, Y27632 (Sigma, Y0503). The cells were incubated at 37 °C in a humidified atmosphere of 5% CO_2_. The medium was changed the next day into the StemFit medium without Y27632. The cells were passaged every 5–6 days (upon 70–80% confluency). The HES7 promoter-luciferase reporter line was described previously^7^. The fluorescent nuclear reporter (pCAG-mCherry-NLS) was introduced into the cells by using a *piggy*Bac vector^58^ and a 4D-Nucleofector (Lonza). After antibiotics selection, a stable clone was isolated. The cells were regularly tested and reported negative for mycoplasma contamination.

### Generation of human somitoids

Ethical approval for this project was granted by Departament de Salut de la Generalitat de Catalunya (Carlos III Program). Once human iPSCs reached 50–70% confluency, the cells were washed with PBS(-) twice and incubated with 2 ml 0.5 mM EDTA in PBS at 37 °C for 6–7 min. The cells were mechanically dissociated by pipetting and transferred into 8 ml of the N2B27 medium described below. The cell suspension was centrifuged at 152 × *g* for 3 min and resuspended into 10 ml of the N2B27 medium. After 1 more wash with the N2B27 medium, the supernatant was completely removed. The cell pellet was resuspended into 150-500 μl of the somitoid induction medium described below with 10 µM Y27632. The cell concentration was adjusted to 7 cells/ml with an adequate amount of the pre-warmed induction medium with Y27632. Then, 50 µl of the cell suspension (=350 cells) was aliquoted into each well of a U-Shaped-Bottom, 96-well-plate (Nunclon Sphera 96U-well plate, Thermo Scientific, 174925) by using a multichannel pipette. The 96-well-plate was centrifuged at 152 × *g* for 2 min for the cells to settle down on the bottom of the plate. One day after aggregate formation, 150 µl of the fresh somitoid induction medium without Y27632 was added to each well. On days 2 and 3, 150 µl of the medium was carefully removed and replaced with 150 µl of the fresh N2B27 medium without disturbing the aggregate. On day 4, 150 µl of the medium was removed from the well and replaced with 150 µl of the fresh N2B27 medium containing 10% Matrigel (growth factor reduced, Corning, 356231). The medium was not changed after the Matrigel addition. The step-by-step instructions and typical problems can be also found in the Protocol Exchange^52^.

### Somitoid induction medium

The N2B27 medium is a mixture of DMEM/F12 (Gibco, 21331020) with 1x N2 supplement (R&D, AR009) and Neurobasal medium (Gibco, 21103049) with 1x B27 supplement (Gibco, 17504-044) at a 1:1 ratio. The N2B27 medium was also supplemented with 2 mM Glutamax (Gibco, 35050-038), 0.1 mM nonessential amino acids (Gibco, 11140-035), 1 mM sodium pyruvate (Gibco, 11360-039), and penicillin/streptomycin (Gibco, 15140-122).

The Somitoid induction medium is the N2B27 medium containing 10 μM SB431542 (Sigma, S4317), 8-10 μM CHIR99021 (Sigma, SML1046), 2 μM DMH1 (Sigma, D8946), and 20 ng/ml bFGF (PeproTech, AF-100-18B).

### Quantitative RT-PCR

Total RNA was extracted with RNeasy Micro Kit (Qiagen, 74004). Then cDNA was synthesized from 0.5 μg of the total RNA with Quantitect Reverse Transcription kit (Qiagen, 205311). Quantitative PCR was performed with LightCycler 480 SYBR Green I Master (Roche, 4707516001) and the gene-specific primers^9,16,59^ (Supplementary Data 2) with LightCycler 480 II (Roche). The expression levels of the target genes were normalized by *GAPDH* expression.

### in situ hybridization chain reaction (HCR)

HCR was performed as described^29^ using probes against the following list of target genes: *HES7* (Accession NM_001165967, hairpin B4), *BRACHYURY* (Accession NM_003181, hairpin B5), *UNCX4.1* (Accession NM_001080461, hairpin B1), *SOX2* (Accession NM_003106, hairpin B3), and *TBX18* (Accession NM_001080508, hairpin B2). Hairpin B1 was labeled with Alexa 546, B2 was labeled with Alexa 488 or Alexa 647, B3 was labeled with Alexa 700, B4 was labeled with Alexa 488, and B5 was labeled with Alexa 647. Images were taken with a MuVi-SPIM Light-Sheet Microscope (Luxendo/Bruker, Luxendo processor software v3.0) The images from the opposing camera views were fused with the Luxendo image processor pipeline using content-based registration.

Analysis of HCR somitoid images was performed using a custom-written napari^60^ plugin which is available via GitHub (Fig. 5f). Images were loaded in the plugin, and the binarized image for each available channel was computed using the Otsu thresholding algorithm. Next, the masks were visually inspected to confirm the accurate segmentation and occasionally correct segmentation errors that were caused, for instance, by the low fluorescence signals in some of the images. To compute the overall volume of the somitoid, we made use of the fact that the genes chosen displayed patterns of expression that jointly covered the whole somitoid structure. An artificial channel which was the sum of all the individual fluorescence channels for the same somitoid was created, and it was binarized using the Otsu algorithm. Given the anisotropic resolution of the 3D stack (Z = 2 μm, XY = 0.391 μm), the positive pixel values within each mask were counted, and the pixel counts were converted into volumes.

### Immunohistochemistry (IHC)

Somitoids were collected with wide-bore tips into 2 ml Eppendorf tubes. After 2 washes with PBS(-), 300 μl of 4% paraformaldehyde (PFA) was applied, and the samples were incubated at 4 °C overnight. After 2 washes with PBS(-), they were further washed twice with the blocking buffer (PBS containing 10% fetal bovine serum (FBS) and 0.2% Triton X-100) for 5 min each. Then, the samples were preincubated in the fresh blocking buffer at room temperature for a few hours before being incubated with an anti-ZO-1 antibody (1/300 dilution, Invitrogen, 61-7300), anti-TBX6 antibody (1/300 dilution, Abcam, ab38883), anti-SOX2 antibody (1/300 dilution, R&D, MAB2018), anti-BRACHYURY antibody (1/300 dilution, R&D, AF2085), anti-SOX1 antibody (1/300 dilution, R&D, AF3369), or anti-PAX6 antibody (1/300 dilution, Abcam, ab195045) in the blocking buffer at 4 °C overnight. The next day, the samples were washed 3 times with the blocking buffer for 15 min each. Then they were incubated with Alexa Fluor 488 Phalloidin (1/300 dilution, Invitrogen, A12379), Alexa Fluor 594 Goat anti-Rabbit IgG (H + L) (1/500 dilution, Invitrogen, A-11037), Alexa Fluor 647 Donkey anti-Goat IgG (H + L) (1/500 dilution, Invitrogen, A32849), Alexa Fluor 647 Goat anti-Mouse IgG (H + L) (1/500 dilution, Invitrogen, A-21236), or Alexa Fluor 488 Goat anti-Mouse IgG (H + L) (1/500 dilution, Invitrogen, A-11029) together with DAPI (1/1000 dilution, Invitrogen, 62247) in the blocking buffer at room temperature for a few hours. The samples were washed 3 times with PBST (0.2% Triton X-100 in PBS) for 15 min each, and images were taken with an FV3000 confocal microscope (Olympus, FV3000 Fluoview RS software), a MuVi-SPIM Light-Sheet Microscope, an LSM 980 Confocal Laser Scanning Microscope (Zeiss, ZEN software), or an Opera Phenix HSC system (PerkinElmer, Harmony software v4.9). When a somitoid spanned multiple images, the tiled images were stitched. The backgrounds of individual images were corrected, and then the images were stitched using ImageJ Grid/Collection stitching so that their backgrounds matched each other.

### EdU labeling

EdU staining was performed using the Click-iT EdU Cell Proliferation Kit (Invitrogen, C10337). Somitoids were collected with wide-bore tips into a 3.5 cm dish and incubated with 10 μM EdU in the N2B27 medium for 2 hrs in a cell culture incubator. The samples were collected with wide-bore tips into a 2 ml Eppendorf tube. After 2 washes with PBS(-), they were stained for SOX2, TBX6, and DAPI according to the IHC protocol described above. After the final step of PBST wash, they were incubated with the Click-iT reaction cocktail at room temperature for 30 min, protected from light. The samples were washed 3 times with PBST for 15 min each, and images were taken with an Opera Phenix HSC system.

### Live imaging of somitoids

Bright-field images of live somitoids were taken with an Opera Phenix HSC system in the wide-field mode using a 10× air objective (NA 0.3). The focus was manually adjusted. For time-lapse imaging, the incubator module was set at 37 °C and 5% CO_2_, and images were taken every 10 min.

### Time-lapse imaging and quantification of HES7 oscillation

The HES7 promoter-luciferase reporter line was used^7^. A glass-bottom 3.5 cm dish was coated with 2% w/v lipidure (Amsbio, CM5206) in ethanol twice at room temperature for 5 min each to prevent somitoids from attaching to the dish. After the second coating, the remaining lipidure solution was removed, and the dish was dried at room temperature until the residual ethanol evaporated. To monitor HES7 oscillations under the microscope (Fig. 3; Supplementary Fig. 12), we put day 4 somitoids on the lipidure-coated dish with 2 ml of the fresh N2B27 medium containing 0.5 mM luciferin and 10% Matrigel. Live-luminescence imaging was performed with an LV200 luminescent microscope (Olympus, cellSens Dimension software) using a 10x objective lens (UPLXAPO10X). Images were taken every 5 or 10 min with 100 ms exposure for the bright field and 1.5 min exposure for the bioluminescence signal. To monitor a collective luciferase signal from day 1 to day 4 (Supplementary Fig. 11a), we collected 8 somitoids and put them on a non-coated 3.5 cm dish with 2 ml of the fresh N2B27 medium containing 0.5 mM luciferin. The luciferase activity was recorded using Kronos Dio Luminometer (Atto, Kronos control software v2.3) every 10 min with 1 min exposure.

For image analyses, the ImageJ software and a Python script were used as previously described^10^. Briefly, kymographs were generated from time-lapse images filtered by Median filter and Tissue aliment filter to keep somitoids at the same position in a frame^61^. Resliced stack images were arranged from top to bottom in a temporal order. The detrended intensity and phase dynamics were calculated by pyBOAT^62^ with a 50-frame window. The peak-to-peak period of HES7 oscillation was measured with Python functions detecting peaks and bottoms of HES7 oscillation. The period of somite formation was manually calculated from time-lapse bright-field images.

### Somite morphometry

DAPI images taken with an FV3000 confocal microscope or an Opera Phenix HSC system were used. Somite morphometric analysis was performed in a semi-automated manner using custom-written Python scripts which is available via GitHub. First, images were loaded and resized to obtain a pixel size of 1 μm and subsequently binarized, choosing an algorithm that could accurately segment the somitoid. The algorithm chosen depends on the pixel intensity histogram, and either the triangle^63^ or the yen^64^ algorithm worked well for all images. Next, holes and debris smaller than 10000 pixels in the binarized image were removed, and the mask was smoothened using the morphological opening operation with a disk-shaped footprint of 5 pixels radius. Then the smoothened mask was used to compute the euclidean distance transform and find its local maxima, which resulted in a disordered collection of 2D points roughly located along the midline of the somitoid. To order the points and find a smooth curve along the longitudinal axis of the somitoid, we manually selected the posterior position and the anterior position of the somitoid. The ordered points were used to generate a B-spline representation with a smoothening value of 10000, and the width of the somitoid at every point along the spline curve was computed. This approach allowed us to generate a characteristic width profile of each somitoid, in which minima and maxima correspond to the somite-to-somite edges and the maximum somite width, respectively (Fig. 1f, top). Somite morphometric parameters were then computed using the minima-to-minima distance (inter-somite distance) and the maxima of the width profile (width) (Fig. 1f, bottom). Somite area and circularity were calculated by using the somite-to-somite distance and the somite width (Supplementary Fig. 3). Only somitoids with single somites were used for this measurement. The graphs were plotted with Python’s boxplot function with the default setting. The middle line of the boxes indicates the median, and the box edges are the 25th and 75th percentiles. The maximum and minimum values are indicated by whiskers.

### Comparison of somite morphometry

Confocal images of somitoids labeled with the fluorescent nuclear reporter were used. The newest somites were manually segmented, and the morphometric parameters were calculated using the ImageJ software. For the somites of human embryos, the images were obtained from the Virtual Human Embryo project^20^ and the Kyoto collection, and the first 4–5 rows of somites were measured. For the somites of mouse trunk-like structures (TLSs) and CHIR99021- and LDN193189-treated TLSs (TLS-CLs)^15^, the DAPI images were provided by the authors. The somites were randomly selected and measured, as the newest somites were difficult to define in TLSs and TLS-CLs. The graphs were plotted with Python’s boxplot function with the default setting. The middle line of the boxes indicates the median, and the box edges are the 25th and 75th percentiles. Values located 1.5 times outside the quartile range were defined as outliers and plotted with dots. The maximum and minimum values, excluding outliers, are indicated by whiskers (Supplementary Fig. 2a).

### Cell morphometry

Phalloidin and DAPI staining image of a day 6 somitoid was taken by a confocal microscope. Nine cells were randomly chosen in 3 regions (Area 1, 2, and 3) of the image, and the cells were manually segmented. The morphometric parameters were calculated using the ImageJ software. The graphs were plotted with Python’s boxplot function with the default setting. The middle line of the boxes indicates the median, and the box edges are the 25th and 75th percentiles. Values located 1.5 times outside the quartile range were defined as outliers and plotted with dots. The maximum and minimum values, excluding outliers, are indicated by whiskers (Fig. 1k).

### Size measurements of somitoids with different initial cell numbers

Somitoids were created from 350 to 1000 human iPSCs that were labeled with the fluorescent nuclear reporter or DAPI, and images were taken by a confocal microscope. The body, which includes NMP and PSM regions, and the newest somites in somitoids were manually segmented. When somitoids showed multiple axes, the longest string of somites was chosen. The morphometric parameters were measured using the ImageJ software. The graphs were plotted with Python’s boxplot function with the default setting. The middle line of the boxes indicates the median, and the box edges are the 25th and 75th percentiles. Values located 1.5 times outside the quartile range were defined as outliers and plotted with dots. The maximum and minimum values, excluding outliers, are indicated by whiskers (Fig. 4f).

### Measurement of first somite formation

Time-lapse bright-field images of somitoids were analyzed using MOrgAna^65^. Briefly, approximately 3% of the images were manually annotated and were fed into MOrgAna to generate a classifier neural network. After the application of the network to the time-lapse images, a segmentation mask, together with morphological parameters, such as area, length, and midline, of the somitoids were obtained for every image frame in the movie (Supplementary Fig. 13a). Subsequently, the left-right (LR) position at which the first somite is formed was annotated manually in the movie. The annotated image frame determined the timing of the first somite formation (Supplementary Fig. 13b). The intersection point P between the LR segment and the midline of the somitoid was used to obtain the relative position of somite formation (Supplementary Fig. 13c). Finally, the angle of the first somite formation was defined as the angle between the vector orthogonal to the LR segment and the tangent vector in the intersecting position P along the midline (Supplementary Fig. 13d).

### Single-cell RNA sequencing (scRNA-seq)

#### Cell preparation

Somitoids were created with individual conditions (Control 10% Matrigel, W/O Matrigel, 3 different concentrations of CHIR) and collected with wide-bore tips into 2 ml Eppendorf tubes on day 7 (Fig. 2; Supplementary Figs. 8–9, 15, 20-22). After 2 washes with PBS(-), the samples were trypsinized into single cells through the treatment with the TrypLE solution and 0.5 mM EDTA in PBS (1:1 mixture) at 37 °C for 5 min. The cells were mechanically dissociated by pipetting and transferred into PBS containing 0.1% bovine serum albumin (BSA). The cell suspension was centrifuged at 200 × *g* for 3 min and resuspended into 0.1% BSA in PBS.

#### MULTI-seq sample preparation

The somitoids created with different CHIR concentrations were labeled with MULTI-seq barcode oligonucleotides for sample multiplexing as described (Supplementary Figs. 20–22)^46^. Briefly, 5 × 10^5^ cells from the prepared single-cell suspension were resuspended in Cell Prep Buffer (PBS(-) containing 0.1% poly(vinyl alcohol) (PVA) and 1 mM EDTA). First, a 1:1 mixture of the cholesterol-conjugated Anchor-oligonucleotides (Anchor CMO, synthesized by Integrated DNA Technologies) and Barcode oligonucleotides with a distinct barcode for each sample (final concentration, 0.2 µM) was added and incubated on ice for 5 min. Then, the same concentration of Co-Anchor CMO (synthesized by Integrated DNA Technologies) was added and incubated for another 5 min, followed by rigorous washes with PBS containing 1% BSA 3 times. After washing, we counted the number of cells in each sample and combined them so that the multiplexed suspension contained the same numbers of cells from each sample. The combined sample was filtered through a 35 µm cell strainer and counted again before barcoding below. The MULTI-seq barcode sequences used in this study are listed in Supplementary Data 3.

#### Barcoding and sequencing

Transcripts of each cell in the single-cell suspension were barcoded with Chromium Controller (10× Genomics, firmware version 4.00). The reagent system used in this study was Chromium Single Cell 3′ GEM, Library & Gel Bead Kit v3 (10× Genomics) and a Chromium Single Cell B Chip Kit (10× Genomics). Barcoding and cDNA library construction were performed according to the manufacturer’s instructions. After the cDNA amplification step, the barcode fraction was collected, amplified, and single-indexed with KAPA HiFi HotStart ReadyMix (Roche, #KK2601) for sequencing. Both finished cDNA and MULTI-seq-barcode libraries were sequenced with NextSeq500 (Illumina). We read 8 base pairs (bp) for TruSeq Indices, 28 bp for 10× barcodes and unique molecular identifiers (UMIs), and 56 bp for both fragmented cDNA and MULTI-seq barcodes.

### scRNA-seq data analyses

#### Read alignment

Sequenced reads were aligned to the human genome (GRCh38) and counted to generate the feature-barcode matrices with the CellRanger pipeline (v.6.1.1, 10× Genomics). The reads having the same UMI were collapsed as a single count. The basic statistics of sequencing results are shown in Supplementary Data 3.

#### Quality control (QC)

The quality control of the single-cell transcriptome data was performed with R version 4.1.2^66^ with the package *Seurat* (v.4.0.5)^67^ as follows: First, we excluded the droplet barcodes that had less than 200 UMIs and the undetected genes that were found less than 3 times in an entire sample. Then, we identified cell barcodes based on scatter plots of detected gene counts against the proportion of mitochondrial gene expression in each cell (Supplementary Fig. 24). The areas demarcated by the red polygons were filtered as cells and used for further analyses. After this initial filtering, potential cell doublets were removed with Scrublet (v.0.2.3)^68^ except the MULTI-seq sample. Doublets in the MULTI-seq sample were removed based on the barcode information. The basic statistics of the filtered cells are shown in Supplementary Data 3.

#### Demultiplexing of the MULTI-seq sample

We counted MUTLI-seq barcode reads associated with the filtered cell barcodes by utilizing the R package *deMULTIplex* (v.1.0.2)^46^, and assigned each cell to one of the following classes: a singlet of one of the original 3 samples, a doublet, or a label-negative cell with *Seurat*. The summary of the demultiplexing results is shown in Supplementary Data 3. We kept only singlets for further analysis.

#### Data normalization

The raw UMI counts of the QC-filtered cells were normalized in each sample according to a deconvolution approach based on pool-based size factors^69^ with the R package *scran* (v.1.22.1)^70^. Briefly, cells were clustered with approximation and pooled as blocks, and the size factor to correct the library size of each cell was calculated with the calculateSumFactors function, taking into account the size factor of the block where the cell belonged. Finally, raw counts of each cell were normalized based on the size factor and Log2-transformed with the logNormCounts function. These scores appear as ‘Log expression level’. To average the expression levels within cell populations, we used the R packages *scuttle* (v.1.4.0)^71^ for heatmaps and *scater* (v.1.22.0)^71^ for dot plots.

#### Identification of highly variable genes (HVGs)

After normalization, highly variable genes were chosen with the modelGeneVarByPoisson function in the *scran* package, which decomposed the total variance of each gene’s expressions into biological and technical components by assuming the Poisson distribution for the technical noise. Genes with the biological variance > 0.1 were chosen as HVGs for further dimension reduction and data integration. The number of HVGs were 1,618, 1,567, and 1,304 for 10% Matrigel, W/O Matrigel, and CHIR MULTI-seq samples, respectively.

#### Data integration and visualization

To integrate all three data sets, we first scaled the normalized data according to the whole library size of each sample with the multiBatchNorm function in the R package *batchelor* (v.1.10.0)^72^. HVGs across data sets were selected as the genes with mean biological variances > 0.1 (*n* = 1,486) for principal component analysis (PCA). We utilized a mutual nearest neighbor approach by fastMNN^72^ in the *batchelor* package for data integration considering the first 20 dimensions of PCA. The corrected results were used for visualization of all samples in a single plot of uniform manifold approximation and projection (UMAP)^73^ with the *Seurat* package. This UMAP embedding was also used for all plots of individual samples simply by splitting the plot sample by sample. The gene expression levels shown in UMAP plots are Log2-transformed normalized values of individual samples or the scaled values with themultiBatchNorm function when multiple samples were involved.

#### Clustering and marker gene detection

To define the cell population clusters, a rank-weighted shared-nearest neighbor graph was constructed for the integrated data with the R *bluster* (v1.4.0)^74^ package, and the Leiden algorithm^75^ was applied for modularity optimization. Marker genes of each cluster were detected with the *scran* package by calculating an area under the curve (AUC) for each gene as an index of the performance to distinguish two clusters in a pairwise comparison, and the minimum AUC against other clusters was taken as a summarizing score of each gene. The genes with the highest minimum AUC were chosen as marker genes of each cluster, and we annotated each cluster based on them and also the expression of other known marker genes. The top 10 markers of each cluster were shown in Supplementary Fig. 8b. This annotation, based on the integrated data set across the three samples, was maintained in the analysis of individual samples to keep consistency in the paper.

#### RNA velocity analysis

Exon/intron read count matrix was generated with Velocyto (v.0.17.17)^76^, and the RNA velocity was calculated and visualized with scVelo (v.0.2.4)^77^.

#### Gene ontology (GO) enrichment analysis

We calculated AUC by comparing the CHIR 5 µM and 10 µM groups and chose 100 upregulated genes with the highest AUC from each group as inputs. The GO enrichment analysis and visualization of the results were performed with the clusterProfiler package (v.4.2.2)^78^ in R.

### Statistics and reproducibility

The number of samples and independent experiments are given in the figure legends. No statistical method was used to predetermine sample size. If cells did not aggregate on day 1 of the somitoid protocol due to cell culture conditions, the experiment was discontinued. The experiments were not randomized. The Investigators were not blinded to allocation during experiments and outcome assessment.

### Reporting summary

Further information on research design is available in the Nature Research Reporting Summary linked to this article.

## Supplementary information

Supplementary Information

Description of Additional Supplementary Files

Supplementary Data 1

Supplementary Data 2

Supplementary Data 3

Supplementary Movie 1

Supplementary Movie 2

Supplementary Movie 3

Supplementary Movie 4

Supplementary Movie 5

Reporting Summary

## Data Availability

The scRNA-seq data used in this study are available in the ArrayExpress database under accession code MTAB-11334. Source data are provided with this paper.

## Source data

Source Data

## Code availability

The custom Python scripts used for the somite morphometry and HCR volume analysis are available from Nikoula86’s Github [github.com/Nikoula86/2022_Somitoids_Analysis].
