# Supplementary material for: Periodic formation of epithelial somites from human pluripotent stem cells
Source: Nat Commun. 2022 Apr 28;13:2325. doi: 10.1038/s41467-022-29967-1 (PMC9050736; doi:10.1038/s41467-022-29967-1)
Supplement: Supplementary file 1 — Supplementary Information [file 41467_2022_29967_MOESM1_ESM.pdf]

Supplementary Information for

Periodic formation of epithelial somites from human pluripotent stem cells

Marina Sanaki-Matsumiya[1*], Mitsuhiro Matsuda[1], Nicola Gritti[1], Fumio Nakaki[1], James Sharpe[1], Vikas Trivedi[1,2], Miki Ebisuya[1*]

[1]European Molecular Biology Laboratory (EMBL) Barcelona, Dr. Aiguader 88, 08003 Barcelona, Spain.

[2]EMBL Heidelberg, Developmental Biology Unit, 69117, Heidelberg, Germany.

*Correspondence to M Sanaki-Matsumiya (marina.matsumiya@embl.es)
or M Ebisuya (miki.ebisuya@embl.es)

- Supplementary Figures 1-24

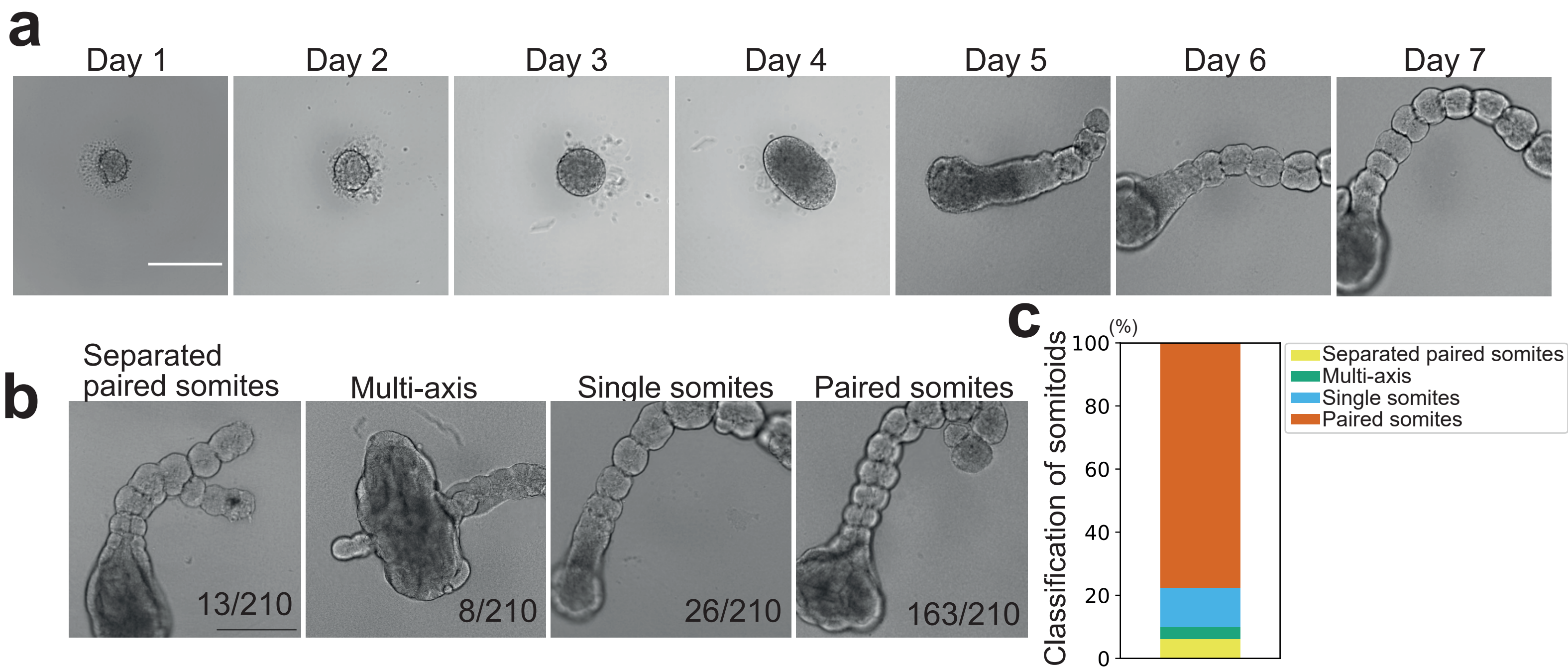

Supplementary Figure 1: Time-course development and classification of somitoids
a, Time-course images of somitoids from day 1 to day 7. Scale bar: 350 µm. **b,** Classification of morphologies of day 7 somitoids. Scale bar: 350 µm. The same images of Single somites, Paired somites, and Separated paired somites are also shown in Fig. 1c. **c,** Graph of the classification. N = 210 from 12 independent experiments. Only the images with the entire somitoid structures were used. Source data are provided as a Source Data file. All images were taken by an Opera Phenix HSC system.

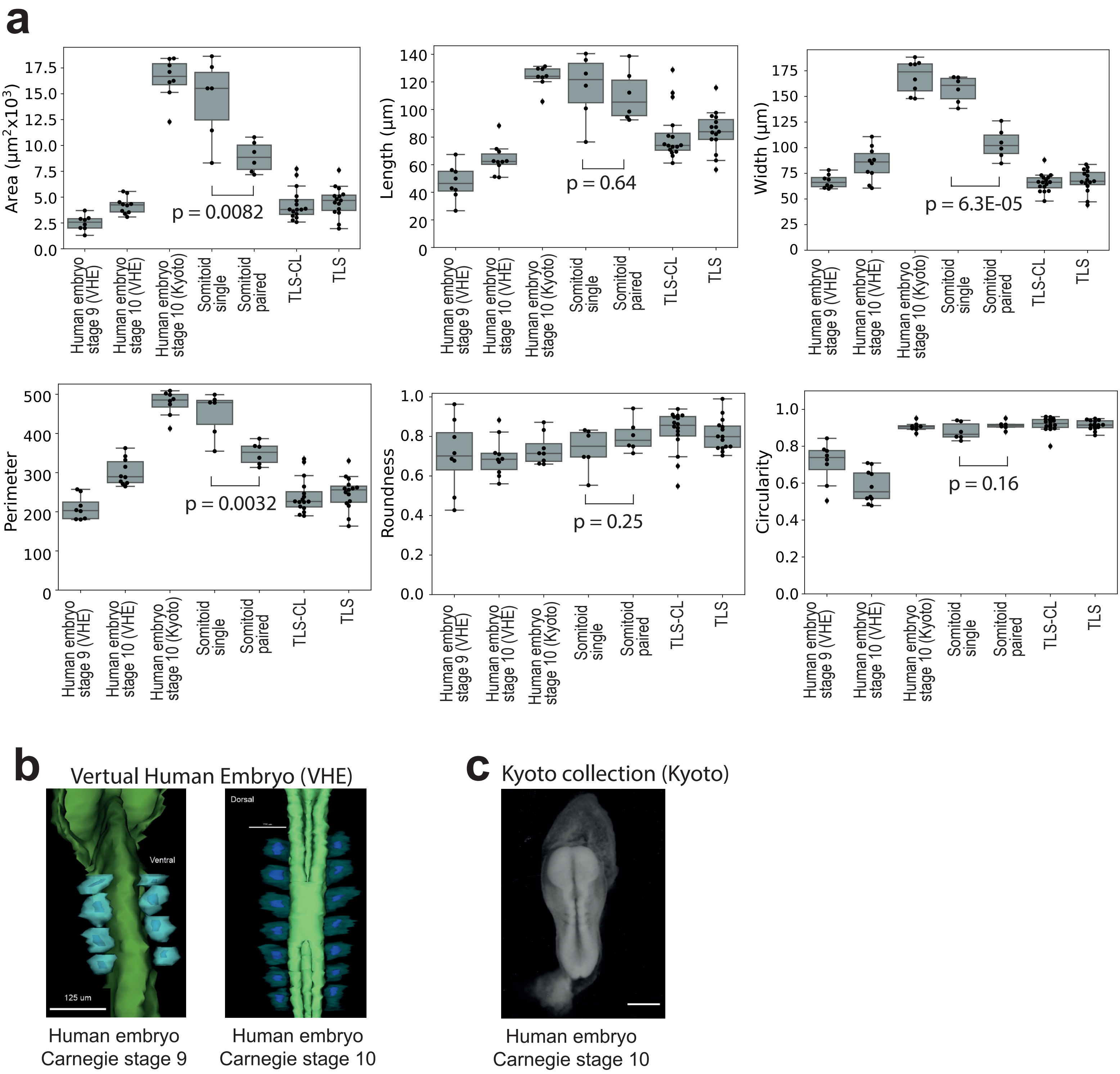

Supplementary Figure 2: Comparison of somite morphometry

a, The size and shape of the newest somites in day 7 somitoids. Paired somites were measured as individual somites. N = 6 (Single somites) and 6 (Paired somites). The images of Carnegie stage 9 and 10 human embryos were obtained from the Virtual Human Embryo (VHE) project and the Kyoto collection #28556 (Kyoto), and the first 4 (VHE stage 9), 5 (VHE stage 10), and 4 (Kyoto) rows of somites were measured. N = 8 (VHE stage 9), 10 (VHE stage 10), and 8 (Kyoto). The data of mouse trunk-like structures (TLSs) and CHIR99021- and LDN193189-treated TLSs (TLS-CLs) were obtained through reanalyzing the images taken by Veenvliet *et al.*, Science, 2020. N = 16 (TLS-CL) and 15 (TLS) randomly selected somites were measured. Boxplots show median, 75th and 25th percentiles, and max and min except for outliers. P-values are from two-sided student's t-test. Source data are provided as a Source Data file. **b,** Images of human embryos from the VHE measured in a. The images are computer-generated images of 3D reconstructions from the serial sections. Three of the reconstructed objects, neural groove/tube, somites, and somitocoel, are shown. The colors were arbitrarily chosen. Scale bars: 125 μm (stage 9) and 114 μm (stage 10). **c,** Original image of the Kyoto collection #28556 measured in a. Scale bar: 500 μm.

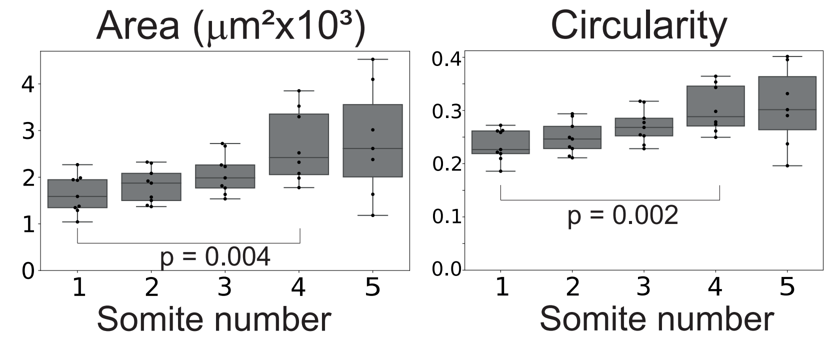

Supplementary Figure 3: Somite circularity along the posterior-anterior axis
Somite area and circularity were calculated by using the somite-to-somite distance and the somite width (Fig. 1f) of day 7 somitoids. Boxplots show median, 75th and 25th percentiles, and max and min. N = 9 (somite number 1-3), 8 (somite number 4), and 7 (somite number 5). P-values are from two-sided paired t-test. Source data are provided as a Source Data file.

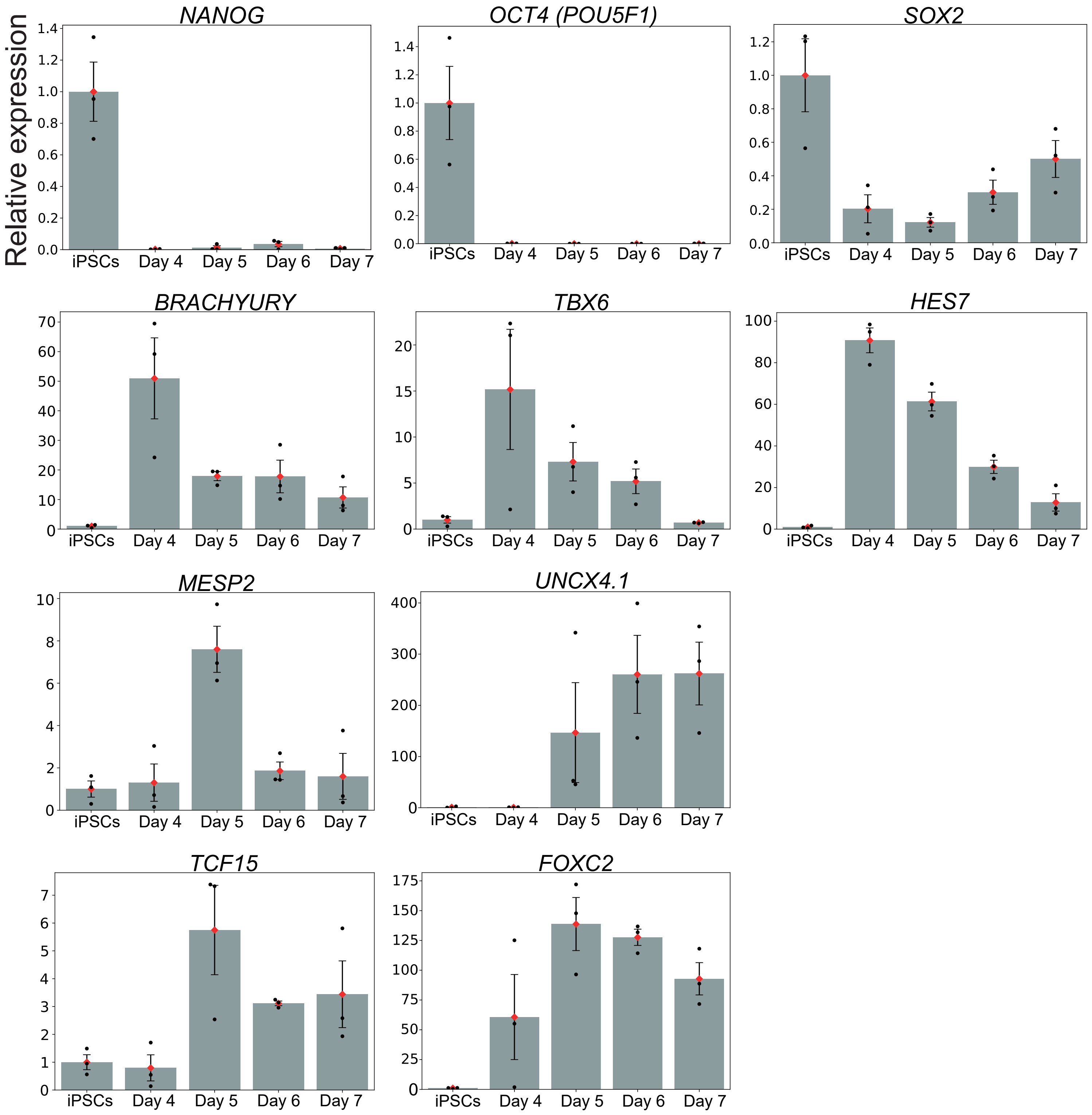

Supplementary Figure 4: qRT-PCR measurements

Relative mRNA levels of selected marker genes in human iPSCs (2D culture) and day 4-7 somitoids. The values were normalized to *GAPDH* expression, and the values of iPSCs were set to 1. Mean ± SEM. N = 3 from 3 independent experiments. Source data are provided as a Source Data file.

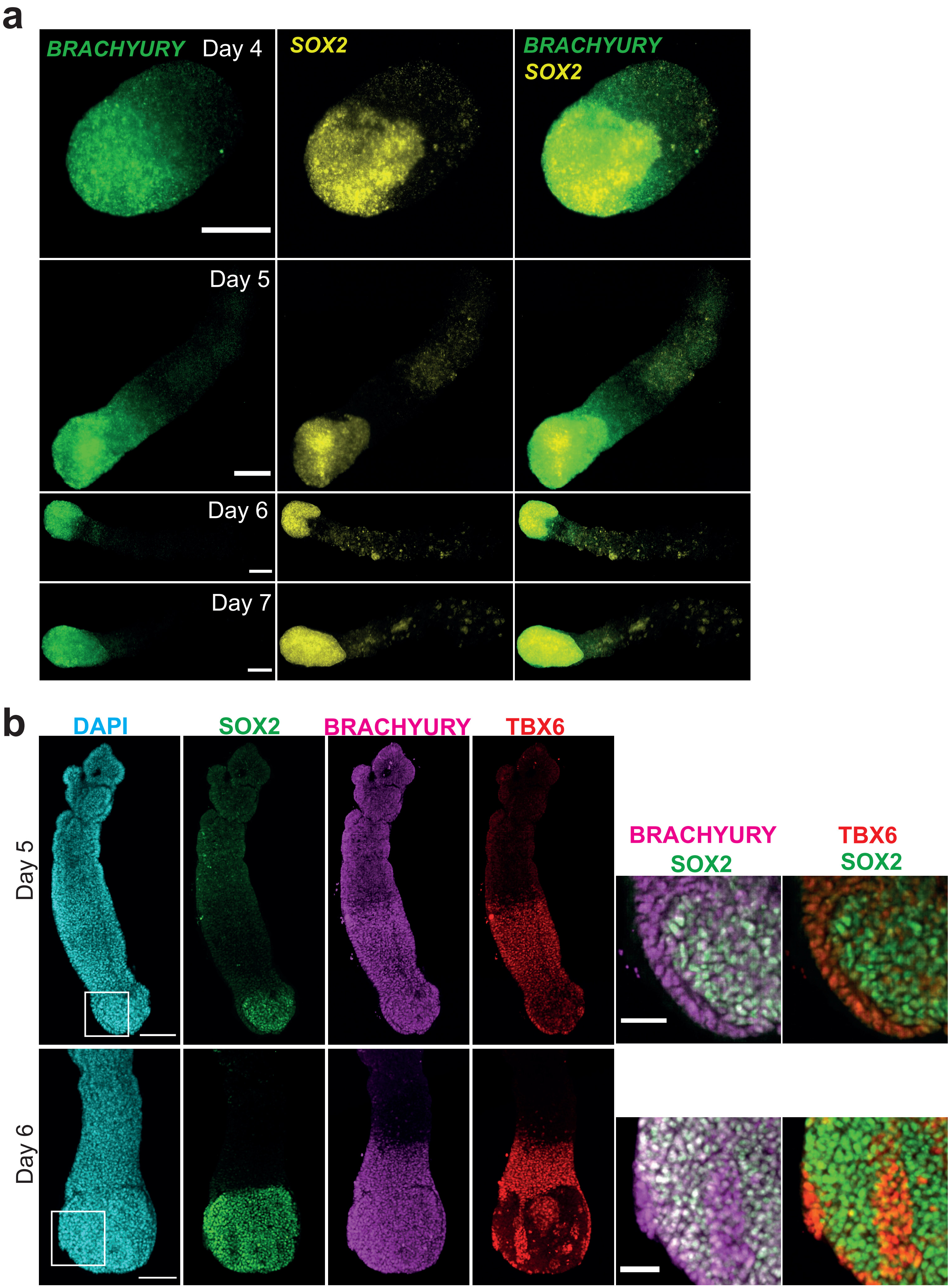
a
BRACHYURY
Day 4
SOX2
BRACHYURY
SOX2
Day 5
Day 6
Day 7
b
DAPI
SOX2
BRACHYURY
TBX6
Day 5
Day 6
BRACHYURY
SOX2
TBX6
SOX2

Supplementary Figure 5: NMPs in somitoids

a, HCR images for *BRACHYURY* and *SOX2* of day 4-7 somitoids. Images were taken by a MuVi-SPIM Light-Sheet Microscope. Scale bars: 150 μm. All samples stained (N = 5 (day 4), 4 (day 5), 6 (day 6), and 3 (day 7)) showed similar expression patterns. **b,** IHC images for SOX2, BRACHYURY, and TBX6 of day 5-6 somitoids. Images were taken by an Opera Phenix HSC system. Scale bars: 150 μm. All samples stained (N = 4 (day 5) and 4 (day 6)) showed similar expression patterns. Enlarged images of the boxed regions are also shown (right). Scale bars: 50 μm.

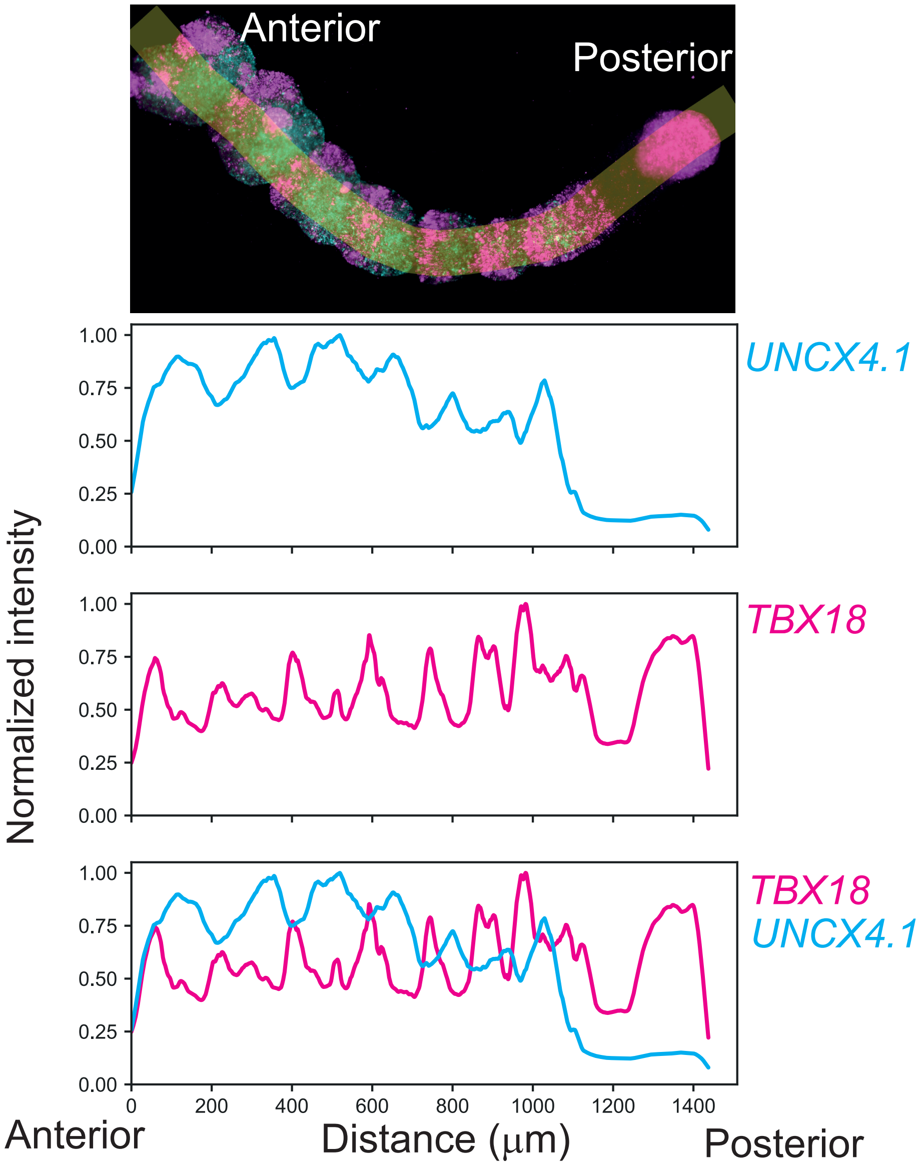

Supplementary Figure 6: Rostral-caudal patterning of somites
Normalized intensities of *UNCX4.1* and *TBX18* of the HCR image shown in Fig. 1g. Source data are provided as a Source Data file.

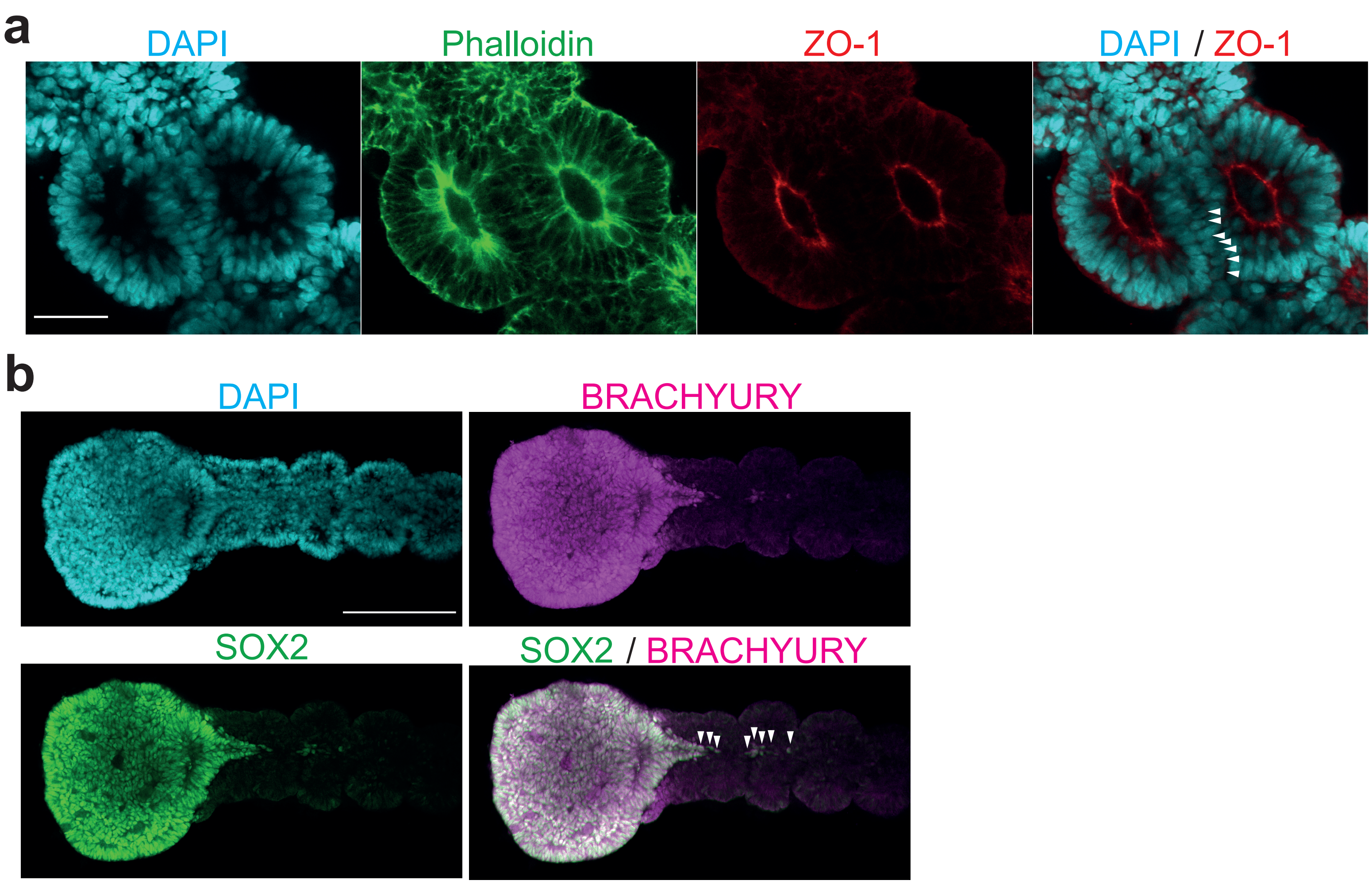

Supplementary Figure 7: Cells between paired somites

a, Representative Phalloidin and ZO-1 staining images of a day 6 paired somitoid. White arrowheads indicate the cells between paired somites. 6/10 samples stained showed similar cells between paired somites. Images were taken by an FV3000 confocal microscope. Scale bar: 50 µm. **b,** Representative IHC images for SOX2 and BRACHYURY of a day 7 paired somitoid. White arrowheads indicate the cells expressing SOX2 and BRACHYURY between paired somites. 2/7 samples stained showed similar NMP-like cells between paired somites. Images were taken by an LSM 980 Confocal microscope. Scale bar: 200 µm.

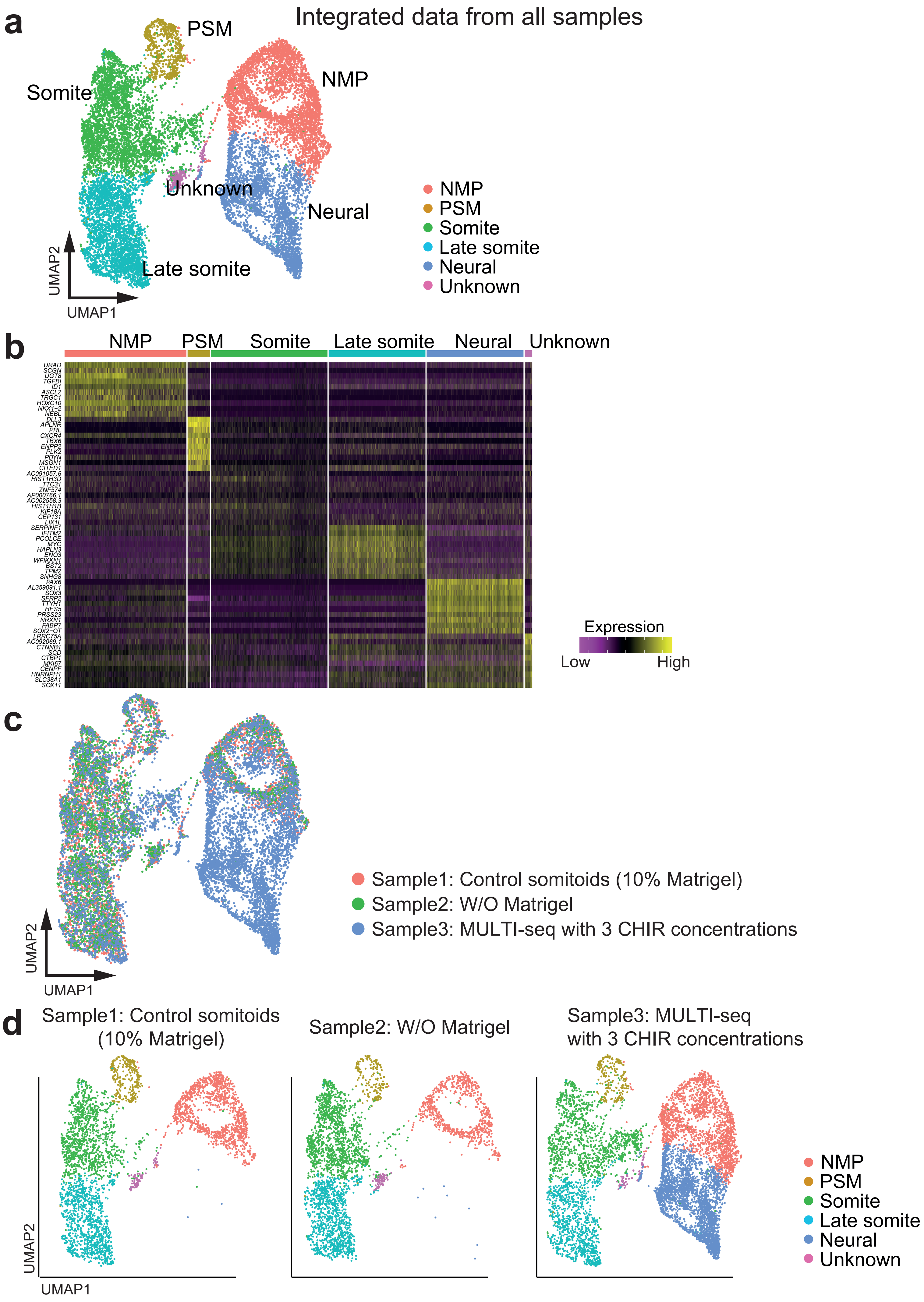
a
Integrated data from all samples
PSM
Somite
NMP
Unknown
Neural
Late somite
UMAP2
UMAP1
NMP
PSM
Somite
Late somite
Neural
Unknown
b
NMP
PSM
Somite
Late somite
Neural
Unknown
URAD
SCGN
UGT8
TGFBI
ID1
ASCL2
TRGC1
HOXC10
NKX1−2
NEBL
DLL3
APLNR
PRL
CXCR4
TBX6
ENPP2
PLK2
PDYN
MSGN1
CITED1
AC091057.6
HIST1H3D
TTC31
ZNF574
AP000766.1
AC002558.3
HIST1H1B
KIF18A
CEP131
LIX1L
SERPINF1
IFITM2
PCOLCE
MYC
HAPLN3
ENO3
WFIKKN1
BST2
TPM2
SNHG8
PAX6
AL359091.1
SOX3
SFRP2
TTYH1
HES5
PRSS23
NRXN1
FABP7
SOX2−OT
LRRC75A
AC092069.1
CTNNB1
SCD
CTBP1
MKI67
CENPF
HNRNPH1
SLC38A1
SOX11
Expression
Low
High
c
UMAP2
UMAP1
Sample1: Control somitoids (10% Matrigel)
Sample2: W/O Matrigel
Sample3: MULTI-seq with 3 CHIR concentrations
d
Sample1: Control somitoids (10% Matrigel)
Sample2: W/O Matrigel
Sample3: MULTI-seq with 3 CHIR concentrations
UMAP2
UMAP1
NMP
PSM
Somite
Late somite
Neural
Unknown

Supplementary Figure 8: scRNA-seq analyses of integrated data

a, Uniform manifold approximation and projection (UMAP) plot of the integrated data from 3 independent samples (Control day 7 somitoids with 10% Matrigel, Somitoids without (W/O) Matrigel, and MULTI-seq samples with 3 CHIR concentrations) used in this study. **b,** Heatmap of 6 identified clusters with the scaled expression of top marker genes. **c,** UMAP plot of the integrated data colored by 3 samples. **d,** Split UMAP plots of individual samples colored by the 6 identified clusters. Note that 'Unknown' is the smallest cluster that represents only 2% of cells. Unlike the 5 other clusters, this cluster was not well-characterized with specific markers and thus named Unknown. Some cells in Unknown express somite markers while others are closer to NMP or neural cells.

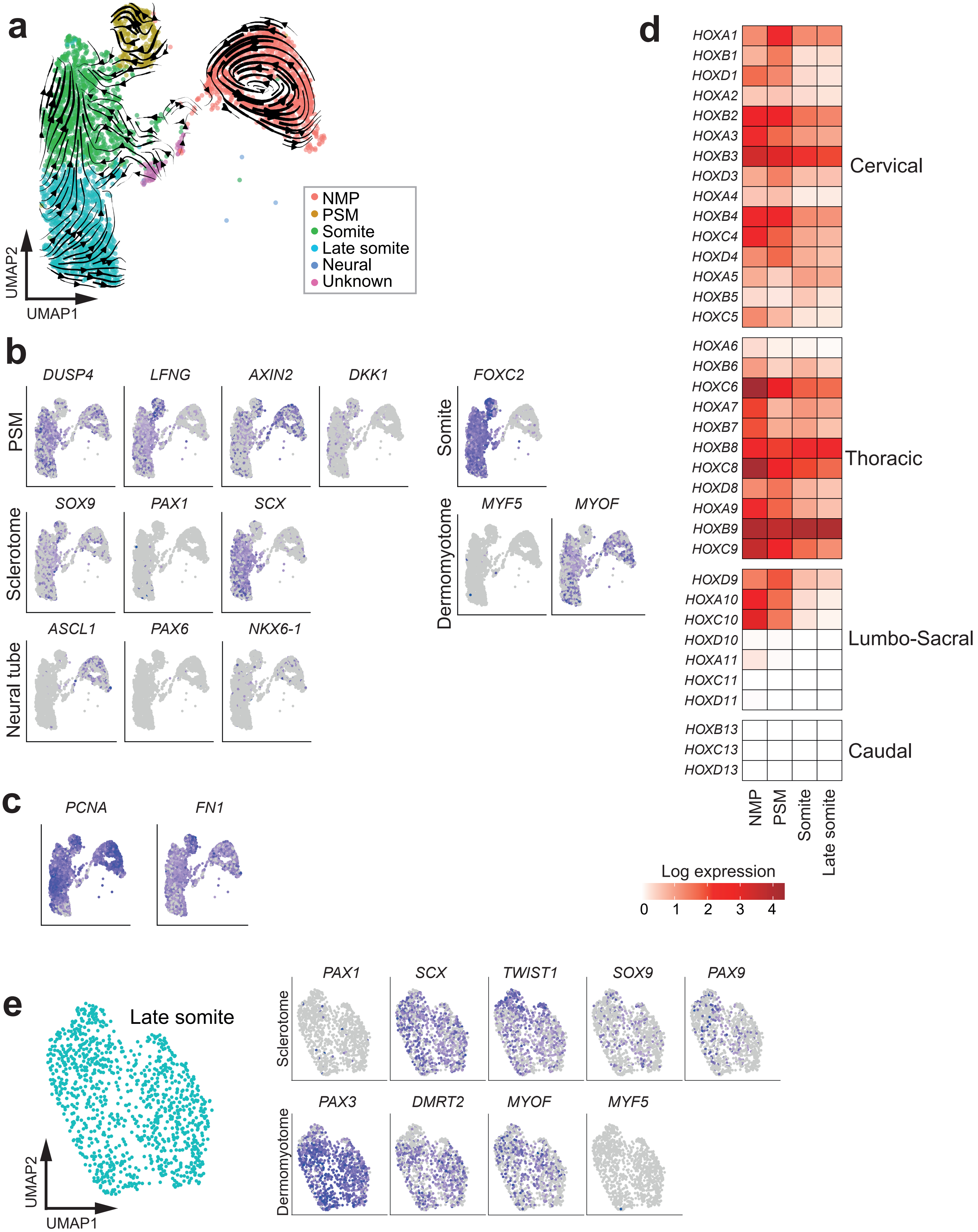

Supplementary Figure 9: scRNA-seq analyses of control somitoids

a, Trajectories inferred from RNA velocity for day 7 somitiods with 10% Matrigel. **b, c,** UMAP plots colored by the expression of indicated genes. **d,** Heatmap of the natural log expression of *HOX* genes. **e,** Subset analysis of Late somite cells shown in Fig. 2d (left). UMAP plots colored by the expression of sclerotome and dermomyotome markers (right).

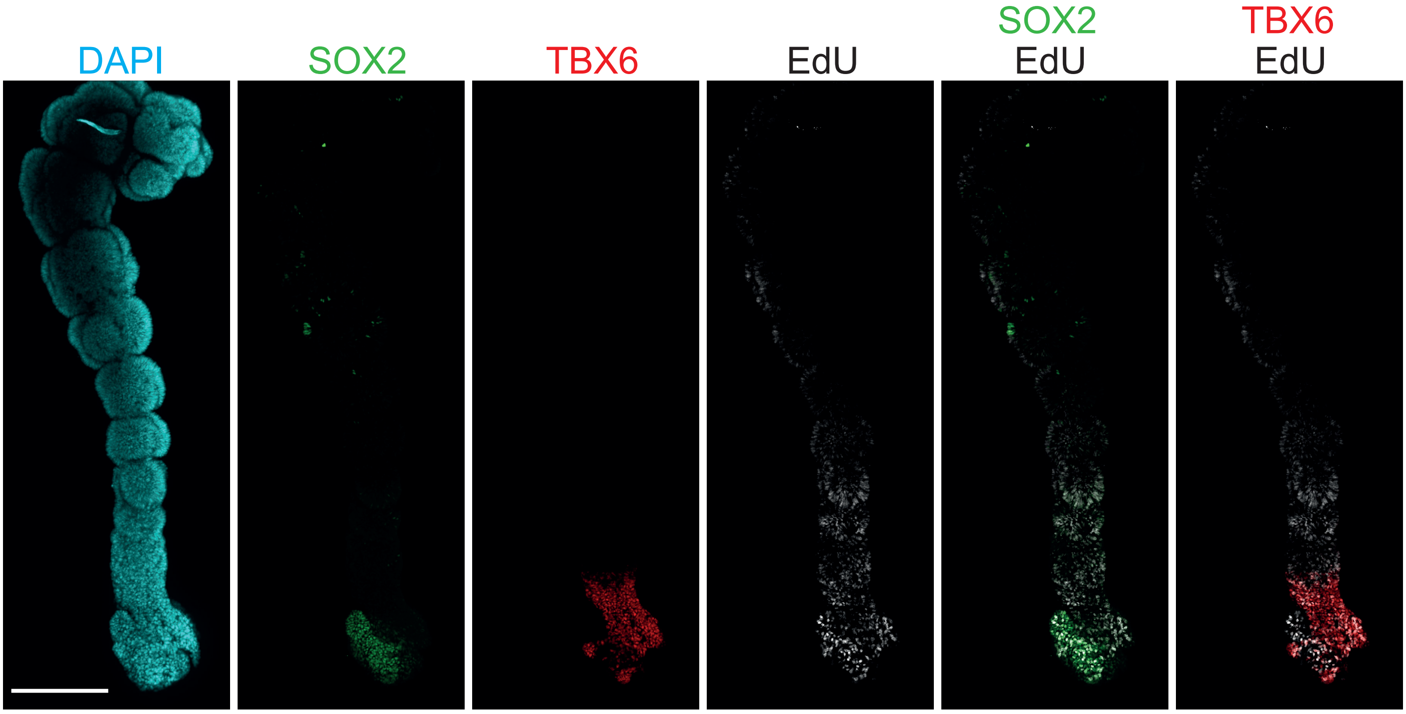

Supplementary Figure 10: Cell proliferation in somitoids
EdU incorporated cells were visualized together with SOX2 and TBX6 staining in day 6 somitoids. Somitoids were incubated in the N2B27 medium containing 10 μM EdU for 2 hrs before fixation. Images were taken by an Opera Phenix HSC system. Scale bar: 300 μm. N = 6 samples showed similar patterns.

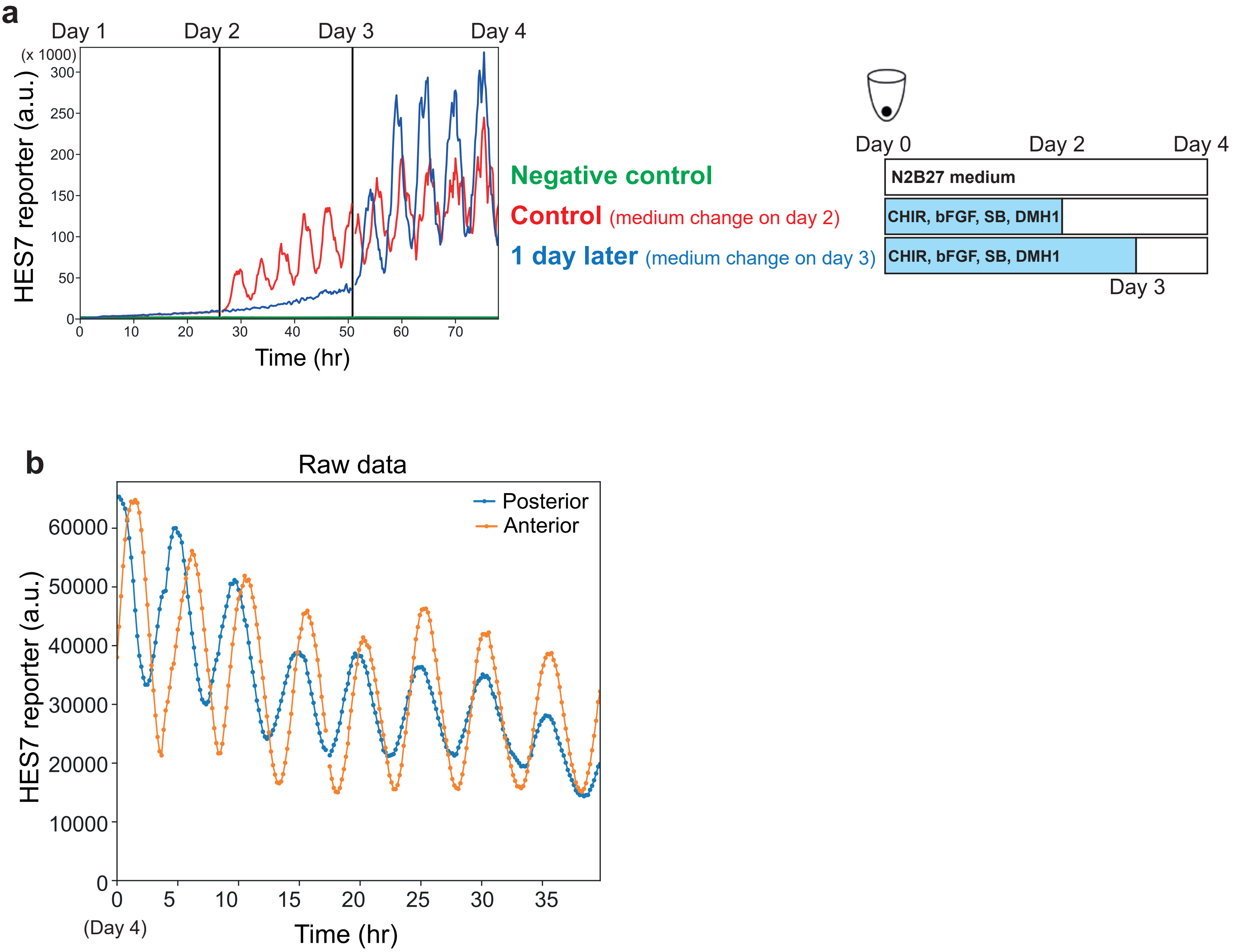

Supplementary Figure 11: Initiation of oscillations

a, The HES7 promoter-luciferase reporter activity in somitoids during days 1-4. Somitoids were treated with CHIR99021, bFGF, SB431542, and DMH1 for 2 days (Control: red) or for 3 days (1 day longer than usual: blue), or not treated (Negative control: green) before the medium change. 8 somitoids were used for each condition in one experiment, and the collective luciferase signal was monitored with a luminometer. Representative graphs of N = 3 samples. 2-3 independent experiments. **b,** Raw data (before detrending) of the HES7 reporter activity of the same sample shown in Fig. 3d. Blue and orange lines indicate the signals measured in posterior and anterior regions of somitoids, respectively, marked in Fig. 3c. The gaps in the graph correspond to short halts of imaging to adjust the sample position. N = 14 samples showed similar oscillatory patterns. Source data are provided as a Source Data file.

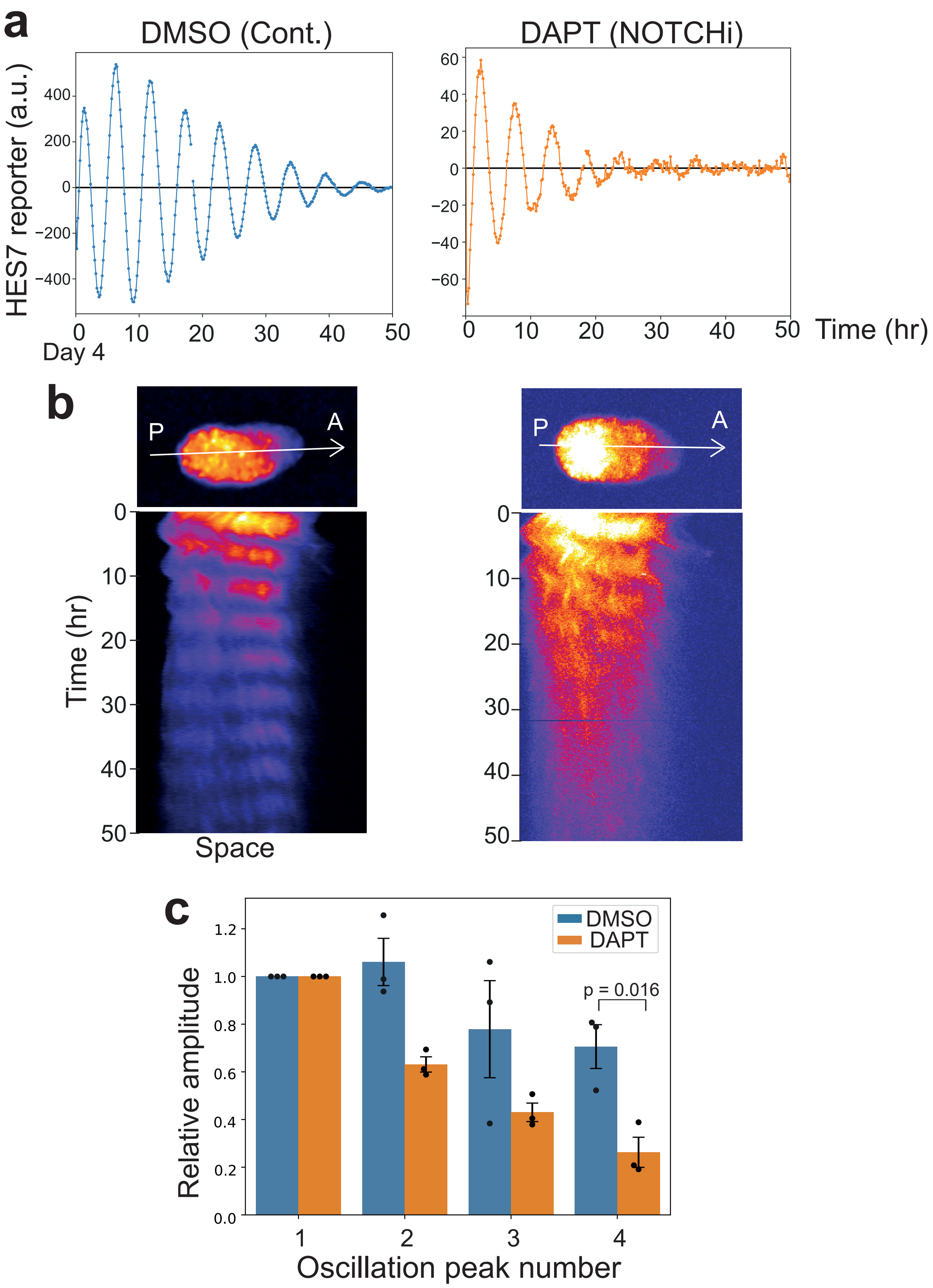

Supplementary Figure 12: Effects of NOTCH inhibition on HES7 oscillations
a, Detrended HES7 reporter activity in the somitoids treated with 10 μM DAPT (NOTCH signaling inhibitor) or DMSO (control) from day 4 onward. The gaps in the graph correspond to short halts of imaging to adjust the sample position. Representative graphs of N = 3 samples. **b,** Kymographs of the HES7 reporter activity measured along the white arrows. P: posterior, A: anterior. **c,** Relative peak amplitudes of oscillation graphs shown in a. The amplitude of each oscillation peak was normalized to that of the first peak. Mean ± SEM. N = 3 from 3 independent experiments. P-value is from two-sided student's t-test. Source data are provided as a Source Data file.

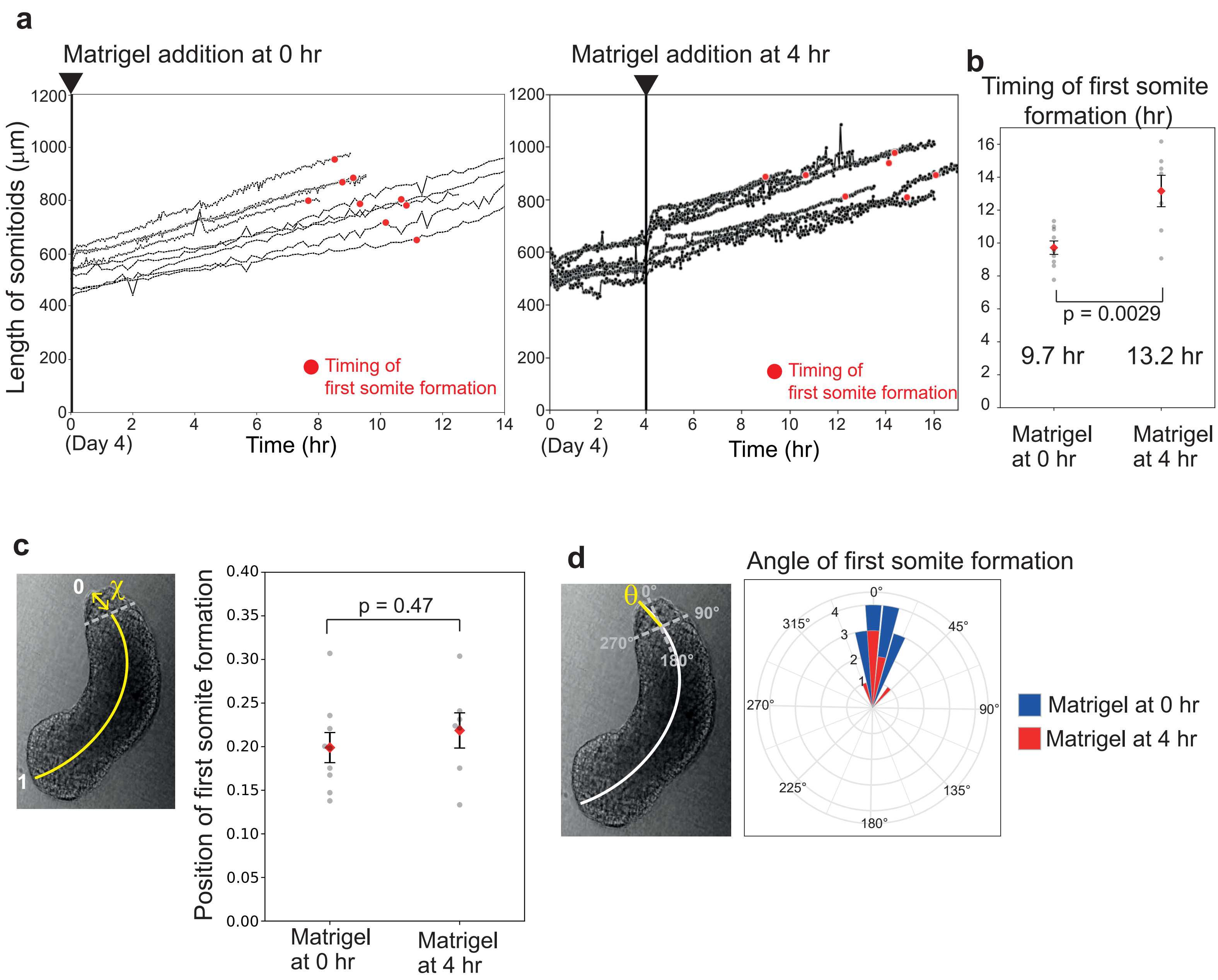

Supplementary Figure 13: Initiation of somite formation

a, Time-course measurements of the somitoid length and the initiation of somite formation. The red dots indicate the timings of the first somite formation. The black arrows indicate the timings of the Matrigel addition on day 4. N = 9 (Matrigel at 0 hr) and 7 (Matrigel at 4 hr) from 5 and 3 independent experiments, respectively. **b,** Timing of the first somite formation calculated from a. Mean ± SEM. P-value is from two-sided student's t-test. **c,** Proportion (χ) of the length of the first somite to the length of the whole somitoid. The whole somitoid length was normalized to 1. Mean ± SEM. P-value is from two-sided student's t-test. **d,** Angle (θ) of the first somite formation with respect to the midline of the somitoid. The somitoid midline was defined as 0 degrees. Source data are provided as a Source Data file.

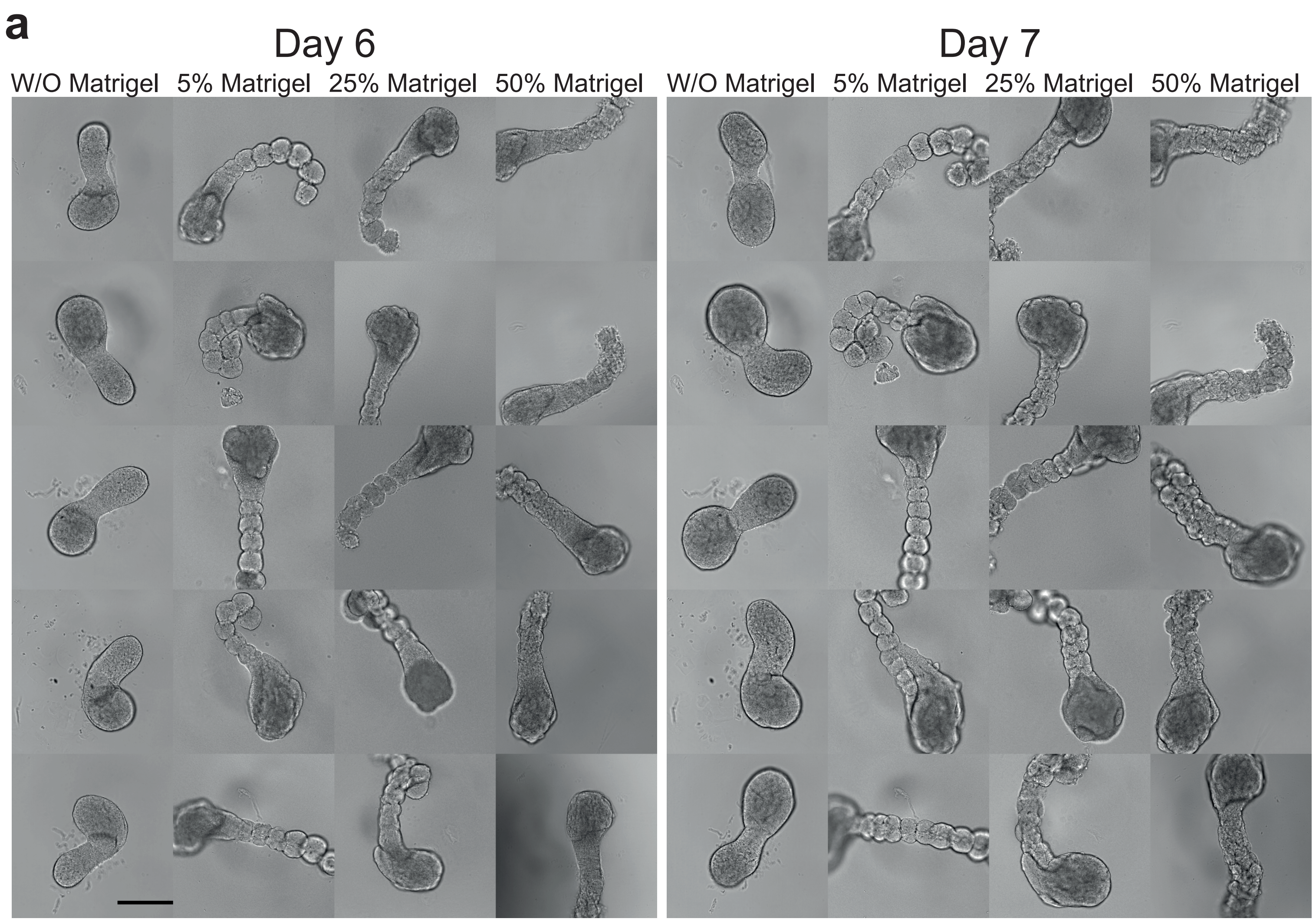

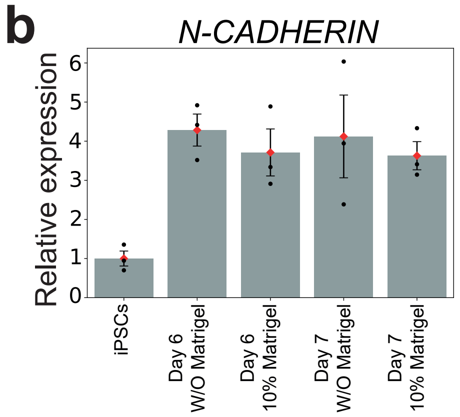

Supplementary Figure 14: Effects of Matrigel concentration

a, Example images of the somitoids created without (W/O) or with (5, 25, and 50%) Matrigel. Matrigel was added on day 4, and the images were taken on day 6 (left) or day 7 (right). Images were taken by an Opera Phenix HSC system. Scale bar: 350 µm. 4 independent experiments. **b,** Relative mRNA levels of *N-CADHERIN* in human iPSCs (2D culture) and the somitoids without or with Matrigel measured by qRT-PCR. The values were normalized to *GAPDH* expression, and the values of iPSCs were set to 1. Mean ± SEM. N = 3 from 3 independent experiments. Source data are provided as a Source Data file.

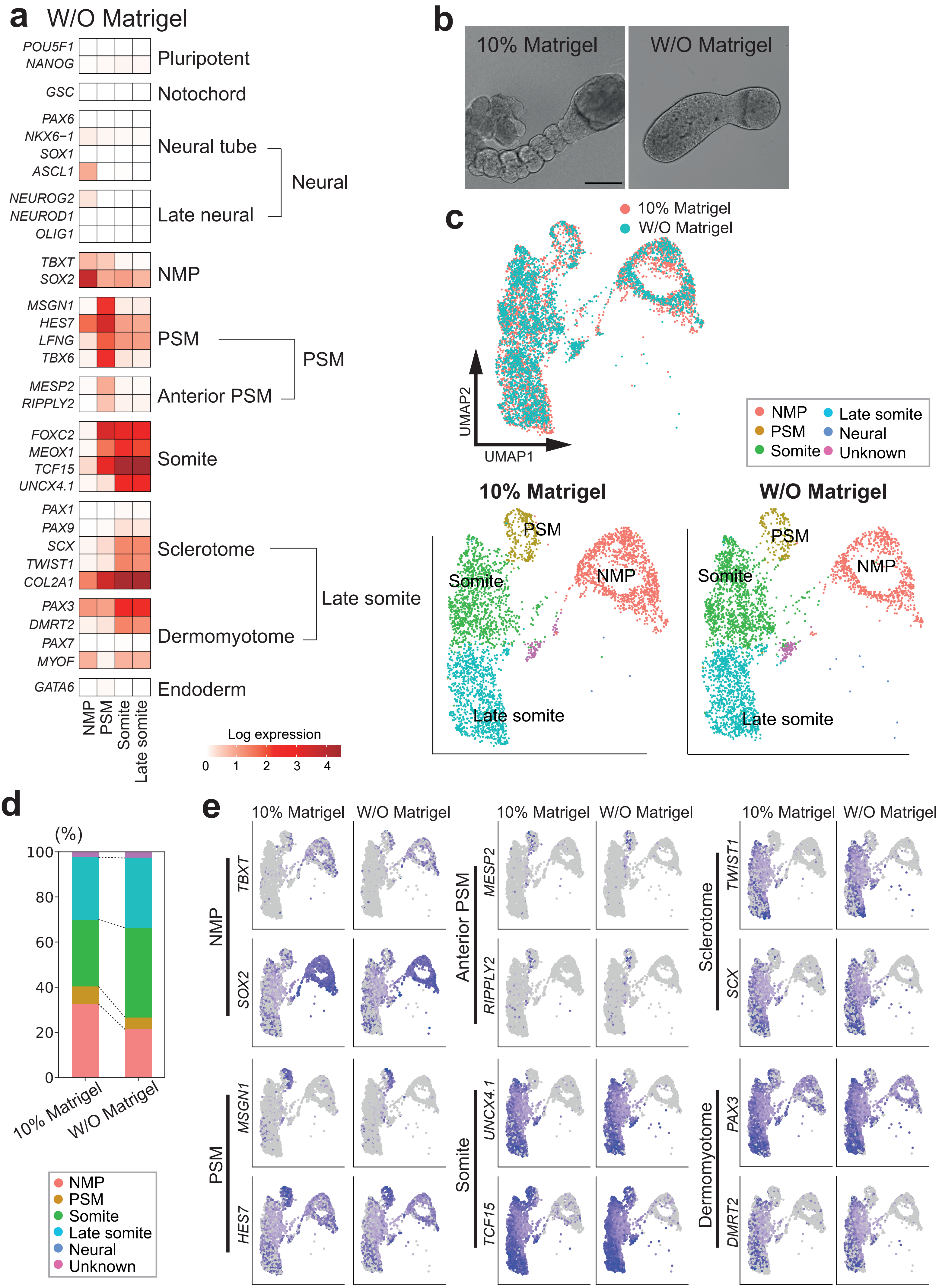
a W/O Matrigel
POU5F1
NANOG
Pluripotent
GSC
Notochord
PAX6
NKX6−1
SOX1
ASCL1
Neural tube
NEUROG2
NEUROD1
OLIG1
Late neural
Neural
TBXT
SOX2
NMP
MSGN1
HES7
LFNG
TBX6
PSM
MESP2
RIPPLY2
Anterior PSM
PSM
FOXC2
MEOX1
TCF15
UNCX4.1
Somite
PAX1
PAX9
SCX
TWIST1
COL2A1
Sclerotome
PAX3
DMRT2
PAX7
MYOF
Dermomyotome
Late somite
GATA6
Endoderm
NMP
PSM
Somite
Late somite
Log expression
0 1 2 3 4
b
10% Matrigel
W/O Matrigel
c
10% Matrigel
W/O Matrigel
UMAP2
UMAP1
NMP
PSM
Somite
Late somite
Neural
Unknown
10% Matrigel
W/O Matrigel
PSM
Somite
NMP
Late somite
d
(%)
100
80
60
40
20
0
10% Matrigel
W/O Matrigel
NMP
PSM
Somite
Late somite
Neural
Unknown
e
10% Matrigel
W/O Matrigel
NMP
TBXT
SOX2
PSM
MSGN1
HES7
Anterior PSM
MESP2
RIPPLY2
Somite
UNCX4.1
TCF15
Sclerotome
TWIST1
SCX
Dermomyotome
PAX3
DMRT2

Supplementary Figure 15: scRNA-seq of somitoids without Matrigel

a, Day 7 somitoids were prepared without (W/O) Matrigel and analyzed by scRNA-seq (N = 29 pooled samples, 3446 cells). Heatmap of the natural log expression of selected marker genes associated with early embryonic development. **b,** Example images of day 7 somitoids with 10% Matrigel (control) and W/O Matrigel. Scale bar: 300 µm. **c,** UMAP plot colored by 10% Matrigel and W/O Matrigel samples (top). 10% Matrigel data is the same as Fig. 2. Split UMAP plots of individual samples colored by the 6 clusters identified in Supplementary Fig. 8a (bottom). The cluster labels are not shown if the number of cells in the cluster is less than 2.5% of the total. **d,** Proportion of the 6 clusters in each sample. Source data are provided as a Source Data file. **e,** UMAP plots colored by the expression of selected marker genes in each sample.

Somitoids from 200 cells

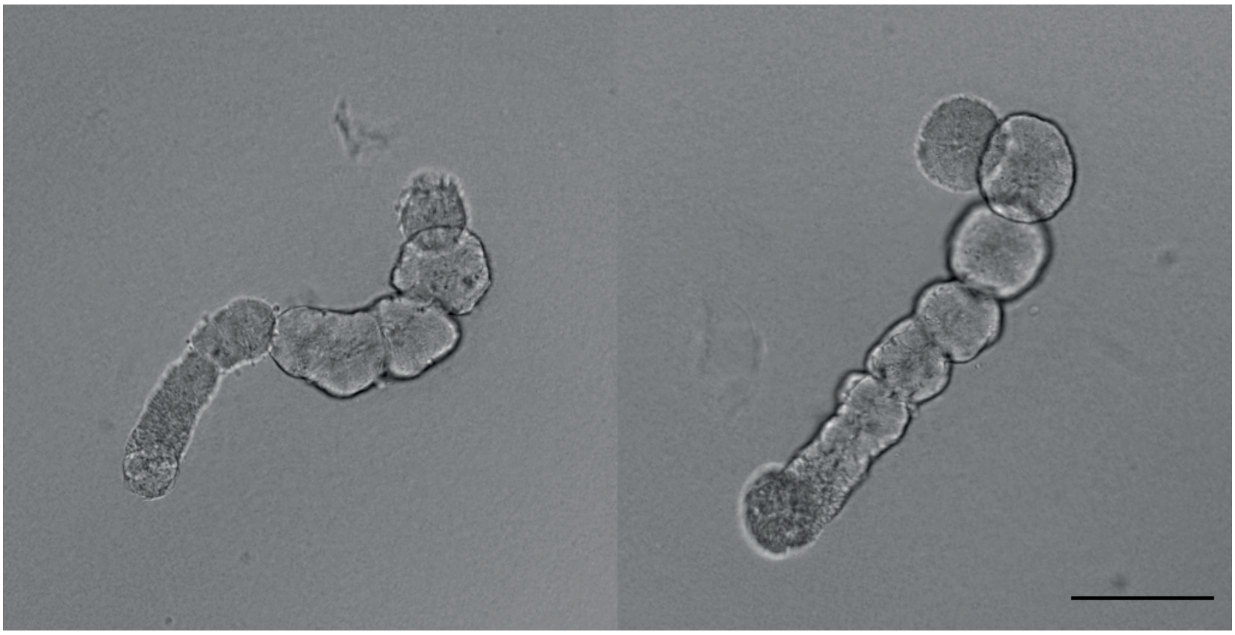

Supplementary Figure 16: Somitoids from a small initial cell number
Bright-field images of the day 6 somitoids created from 200 cells instead of the usual 350 cells. All images were taken by an Opera Phenix HSC system. Scale bar: 200 µm. Representatives of N = 21 samples. 3 independent experiments.

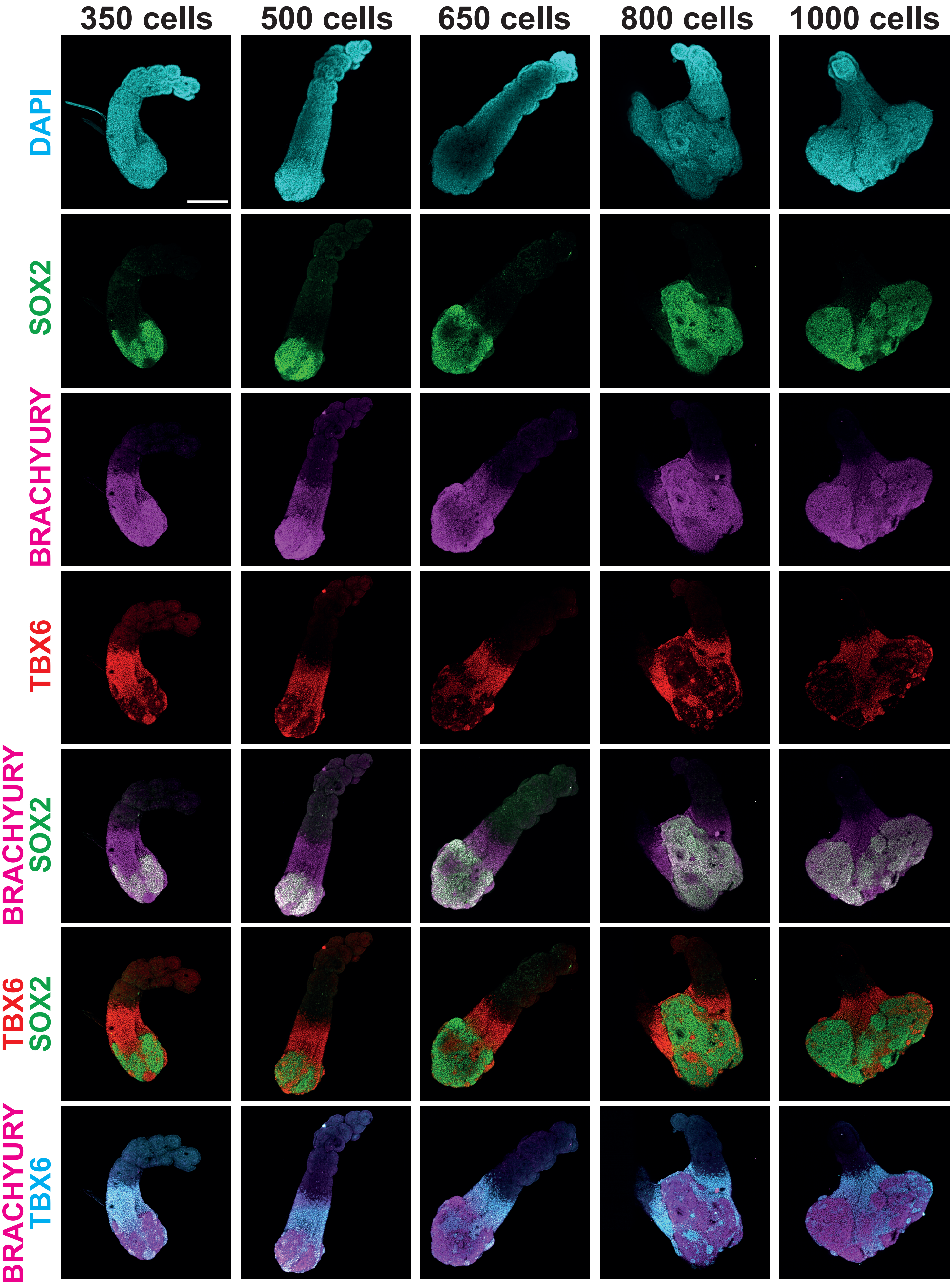

Supplementary Figure 17: NMP and PSM regions in the somitoids with varying initial cell numbers
IHC images for SOX2, BRACHYURY, and TBX6 of the day 5 somitoids created with different initial cells numbers. Images were taken by an Opera Phenix HSC system. Scale bar: 300 μm. All samples stained (N = 4 (350 cells), 13 (500 cells), 12 (650 cells), 10 (800 cells), and 14 (1000 cells)) showed similar expression patterns.

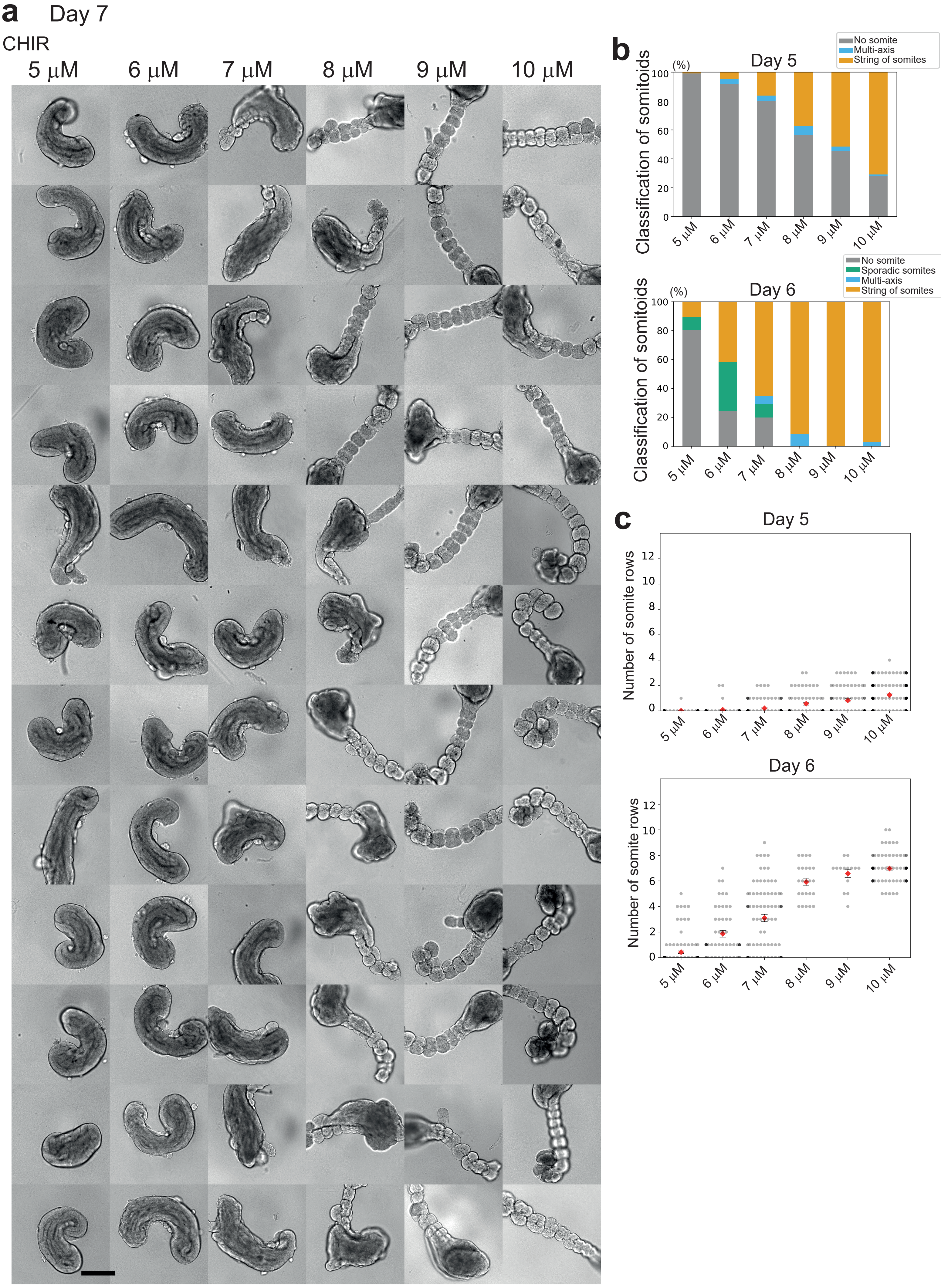
a
Day 7
CHIR
5 μM
6 μM
7 μM
8 μM
9 μM
10 μM
b
Day 5
(%)
Classification of somitoids
No somite
Multi-axis
String of somites
0
20
40
60
80
100
5 μM
6 μM
7 μM
8 μM
9 μM
10 μM
Day 6
No somite
Sporadic somites
Multi-axis
String of somites
c
Day 5
Number of somite rows
0
2
4
6
8
10
12
Day 6

Supplementary Figure 18: Effects of CHIR dosage on somitoids

a, Example images of the somitoids created with different CHIR concentrations. CHIR was added during the initial 2 days and then washed out, and the images were taken on day 7. Images were taken by an Opera Phenix HSC system. Scale bar: 350 µm. **b,** Classification of somitoids on day 5 (top) and day 6 (bottom). Sporadic somites mean only 1-2 isolated somites, whereas strings of somites mean more than 3 rows of somites. N of day 5 samples = 124 (5 µM), 61 (6 µM), 124 (7 µM), 62 (8 µM), 68 (9 µM), and 178 (10 µM). 7-15 independent experiments. N of day 6 samples = 107 (5 µM), 53 (6 µM), 110 (7 µM), 36 (8 µM), 36 (9 µM), and 155 (10 µM). 3-11 independent experiments. **c,** Quantification of the number of somite rows on day 5 (top) and day 6 (bottom). Mean ± SEM. N of day 5 samples = 118 (5 µM), 60 (6 µM), 123 (7 µM), 59 (8 µM), 65 (9 µM), and 160 (10 µM). 7-15 independent experiments. N of day 6 samples = 106 (5 µM), 51 (6 µM), 82 (7 µM), 23 (8 µM), 14 (9 µM), and 75 (10 µM). 3-11 independent experiments. Only the images with the entire somitoid structures were measured. Part of samples of 8-10 µM is common to Fig. 1e. **b, c,** For classification and quantification of day 7 somitoids, see Fig. 5c,d. Source data are provided as a Source Data file.

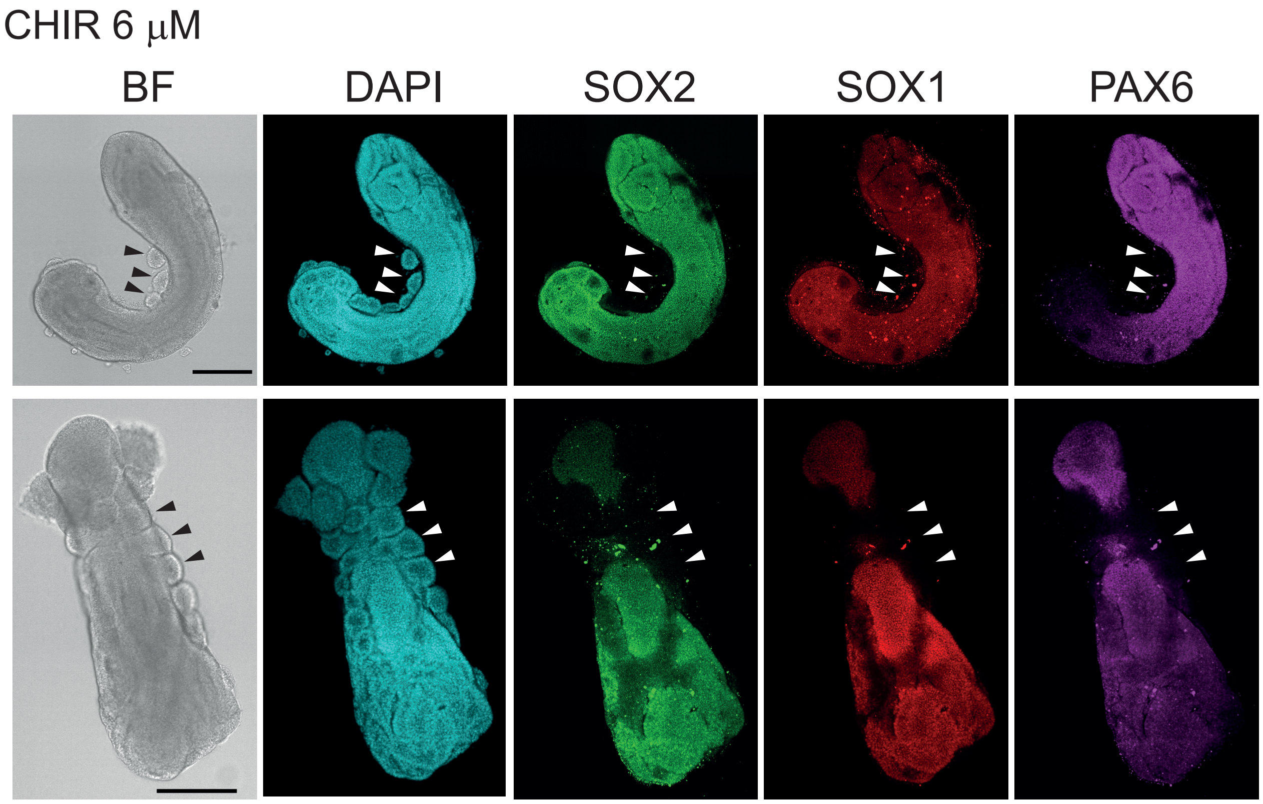

Supplementary Figure 19: Neural tube markers in the somitoids created with a lower CHIR dose
IHC images for neural tube markers (SOX2, SOX1, and PAX6) of the day 7 somitoids created with 6 μM CHIR. Arrowheads indicate somites. Images were taken by an Opera Phenix HSC system. Scale bars: 300 μm. N = 3 samples showed similar expression patterns.

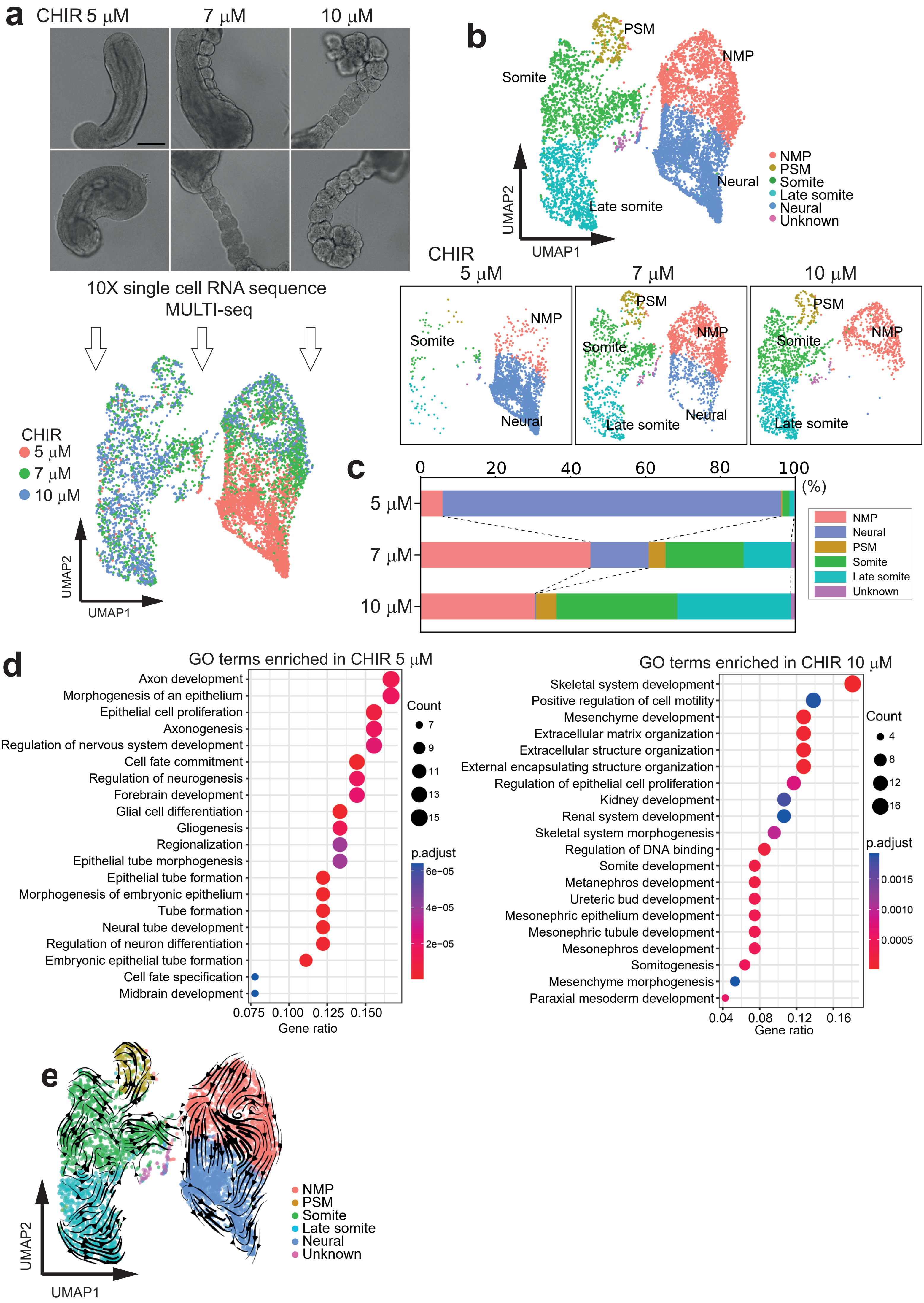
a
CHIR 5 μM
7 μM
10 μM
10X single cell RNA sequence
MULTI-seq
CHIR
5 μM
7 μM
10 μM
UMAP2
UMAP1
b
PSM
NMP
Somite
Neural
Late somite
NMP
PSM
Somite
Late somite
Neural
Unknown
UMAP2
UMAP1
CHIR
5 μM
7 μM
10 μM
c
0 20 40 60 80 100
(%)
5 μM
7 μM
10 μM
NMP
Neural
PSM
Somite
Late somite
Unknown
d
GO terms enriched in CHIR 5 μM
Axon development
Morphogenesis of an epithelium
Epithelial cell proliferation
Axonogenesis
Regulation of nervous system development
Cell fate commitment
Regulation of neurogenesis
Forebrain development
Glial cell differentiation
Gliogenesis
Regionalization
Epithelial tube morphogenesis
Epithelial tube formation
Morphogenesis of embryonic epithelium
Tube formation
Neural tube development
Regulation of neuron differentiation
Embryonic epithelial tube formation
Cell fate specification
Midbrain development
0.075 0.100 0.125 0.150
Gene ratio
Count
7
9
11
13
15
p.adjust
6e−05
4e−05
2e−05
GO terms enriched in CHIR 10 μM
Skeletal system development
Positive regulation of cell motility
Mesenchyme development
Extracellular matrix organization
Extracellular structure organization
External encapsulating structure organization
Regulation of epithelial cell proliferation
Kidney development
Renal system development
Skeletal system morphogenesis
Regulation of DNA binding
Somite development
Metanephros development
Ureteric bud development
Mesonephric epithelium development
Mesonephric tubule development
Mesonephros development
Somitogenesis
Mesenchyme morphogenesis
Paraxial mesoderm development
0.04 0.08 0.12 0.16
Gene ratio
Count
4
8
12
16
p.adjust
0.0015
0.0010
0.0005
e
NMP
PSM
Somite
Late somite
Neural
Unknown
UMAP2
UMAP1

Supplementary Figure 20: scRNA-seq of somitoids with varying amounts of WNT signaling

a, Example images of the day 7 somitoids created with 3 different CHIR concentrations. Scale bar: 300 μm. Somitoids (N = 25 (CHIR 5 μM), 30 (7 μM), and 23 (10 μM) pooled samples; 2945, 2453, and 1933 cells, respectively) were collected and labeled with MULTI-seq barcodes. After MULTI-seq analyses, the UMAP was colored by the barcodes corresponding to individual CHIR conditions. **b,** UMAP plot of the integrated data from all 3 CHIR conditions colored by the 6 clusters identified in Supplementary Fig. 8a (top). Split UMAP plots of individual conditions (bottom). The cluster labels are not shown if the number of cells in the cluster is less than 1.5% of the total. **c,** Proportion of the 6 clusters in each CHIR condition. Source data are provided as a Source Data file. **d,** Gene Ontology (GO) analyses. GO terms overrepresented in the top 100 genes upregulated by 5 μM CHIR as compared with 10 μM CHIR (left). GO terms overrepresented in the top 100 genes upregulated by 10 μM CHIR as compared with 5 μM CHIR (right). The top 100 genes are listed in Supplementary Data 1. **e,** Trajectories inferred from RNA velocity.

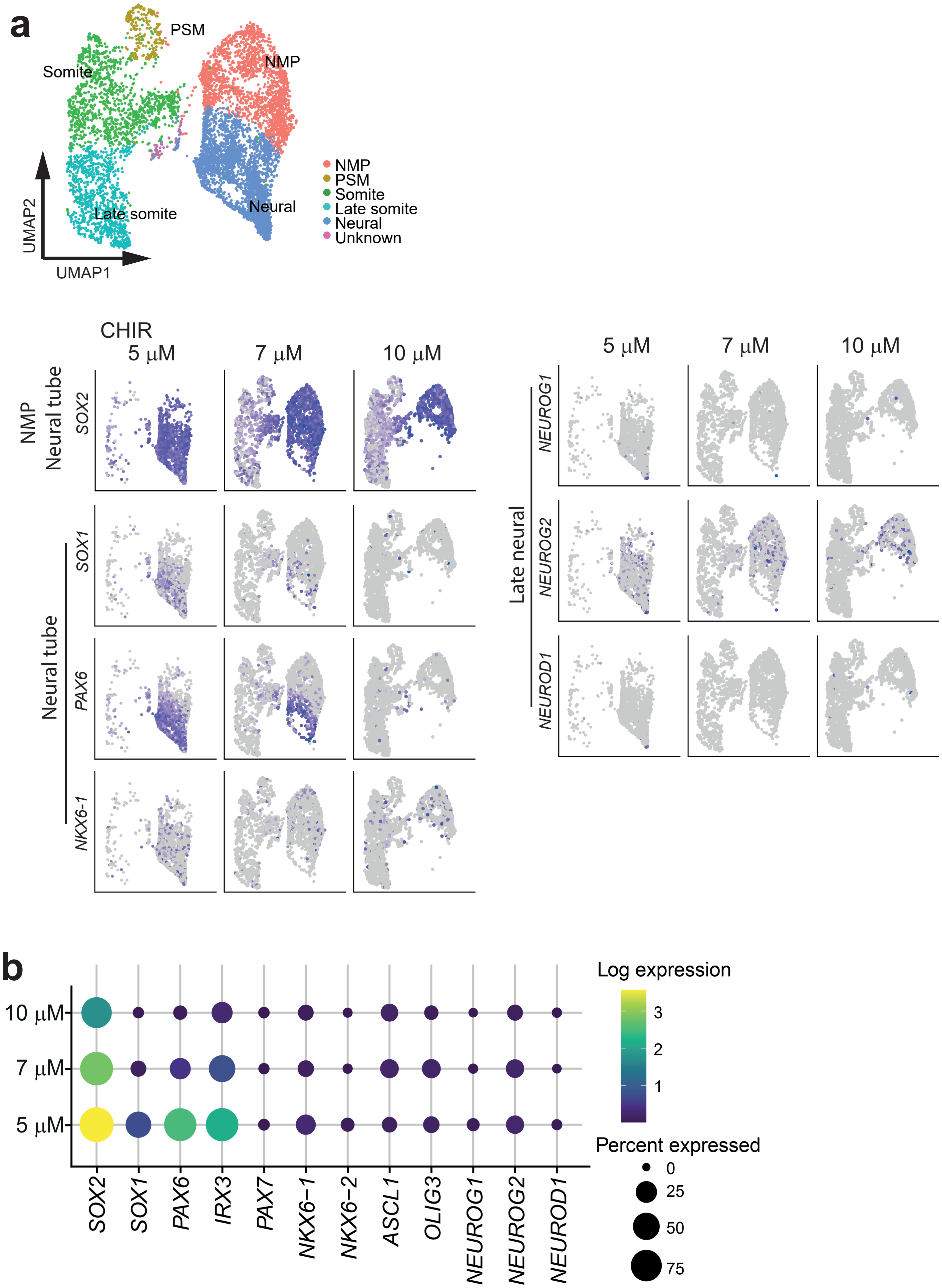

Supplementary Figure 21: Effects of CHIR dosage on neural lineages

a, UMAP plots colored by the expression of selected marker genes for neural lineages in each CHIR condition. **b,** Dot plot of the natural log expression of marker genes for neural lineages.

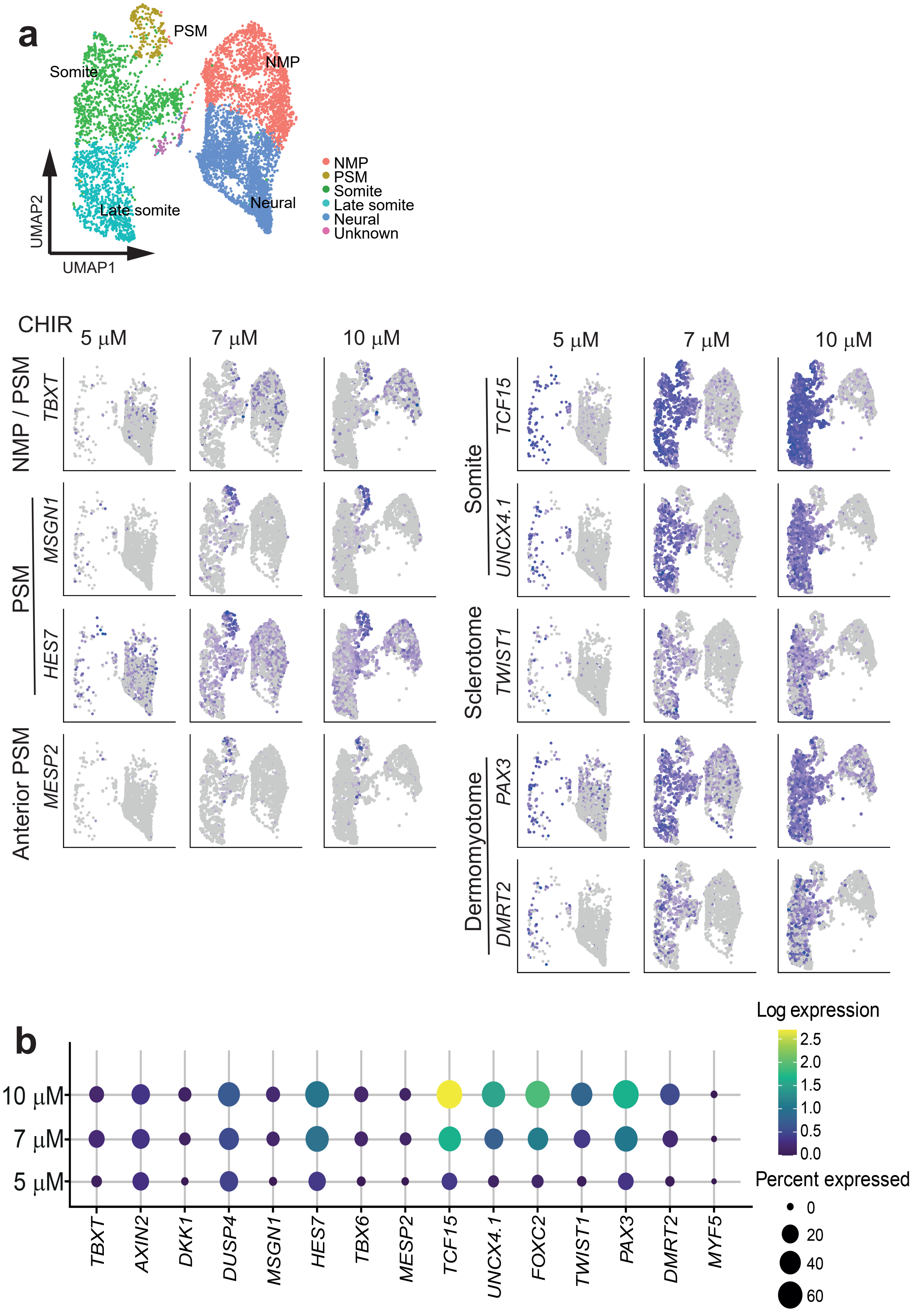

Supplementary Figure 22: Effects of CHIR dosage on mesodermal lineages

a, UMAP plots colored by the expression of selected marker genes for mesodermal lineages in each CHIR condition. **b,** Dot plot of the natural log expression of marker genes for mesodermal lineages.

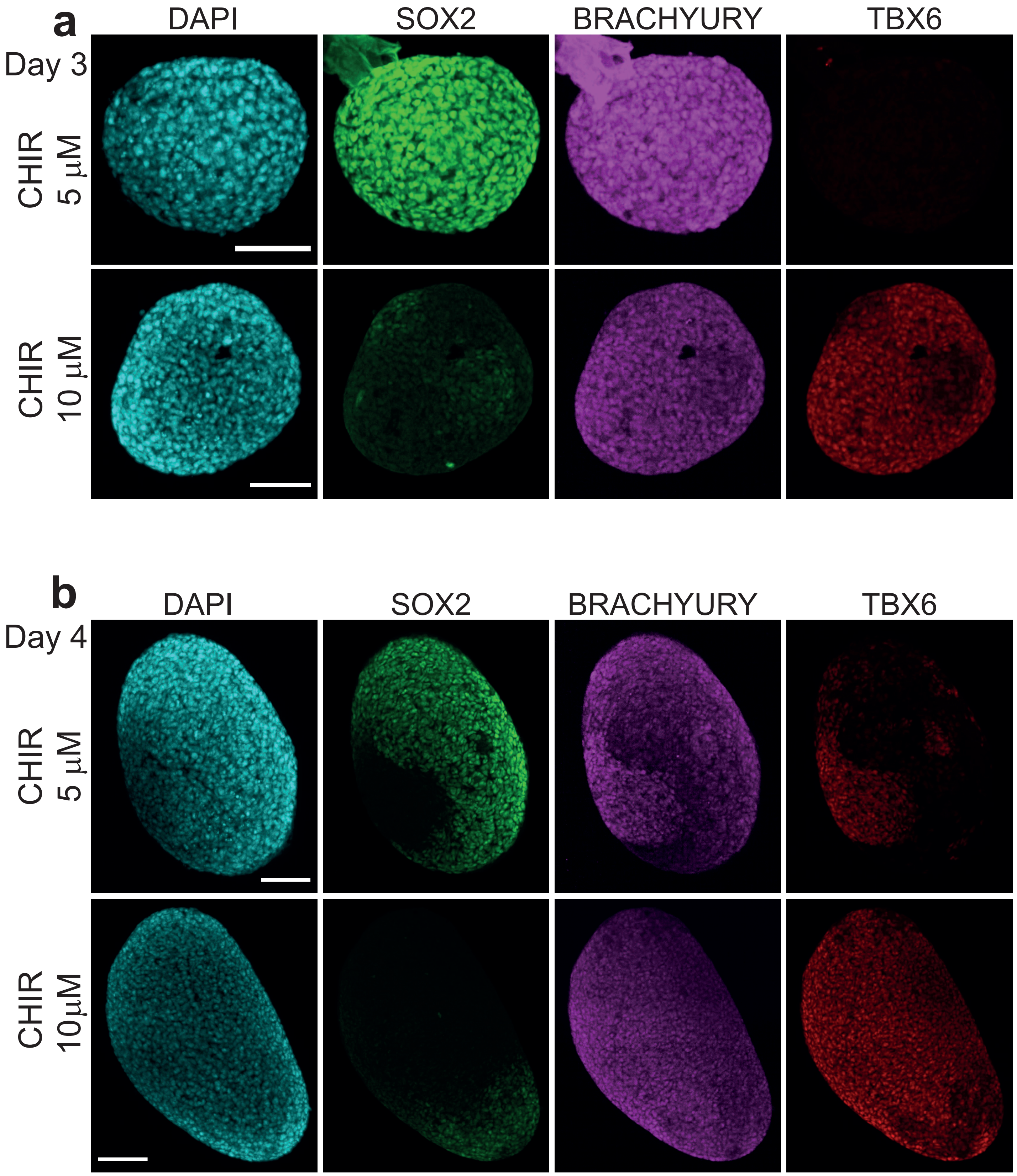

Supplementary Figure 23: NMP and PSM markers in early-stage somitoids
a, IHC images for SOX2, BRACHYURY, and TBX6 of the day 3 somitoids created with 5 μM or 10 μM CHIR. All samples stained (N = 7 (5 μM) and 10 (10 μM)) showed similar expression patterns. **b,** IHC images for SOX2, BRACHYURY, and TBX6 of the day 4 somitoids created with 5 μM or 10 μM CHIR. All samples stained (N = 11 (5 μM) and 8 (10 μM)) showed similar expression patterns. All images were taken by an Opera Phenix HSC system. Scale bars: 100 μm.

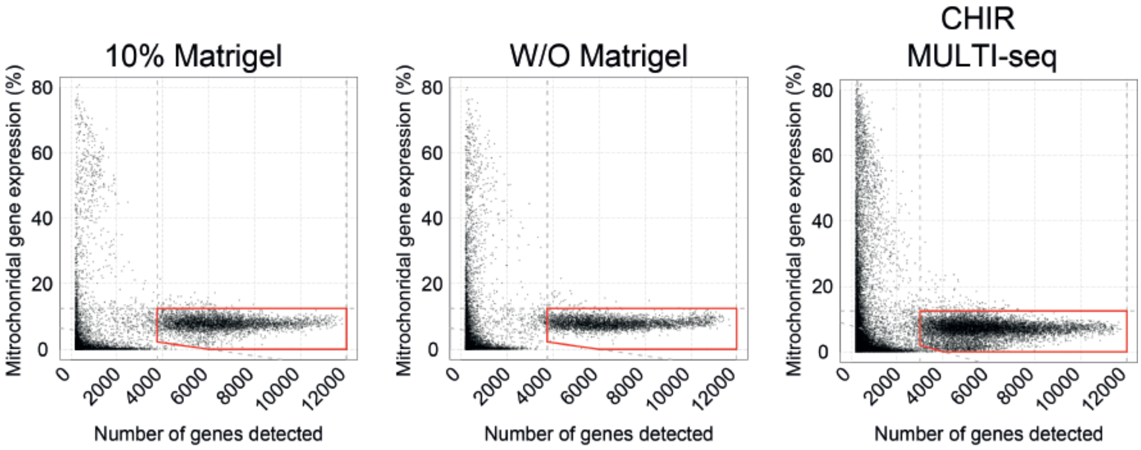

Supplementary Figure 24: Quality control data for scRNA-seq
Scatter plots for the quality control of the scRNA-seq datasets. The areas demarcated by the red polygons were filtered as cells and used for further analyses. See the Methods section for details.
