# Supplementary material for: Periodic formation of epithelial somites from human pluripotent stem cells
Source: Nat Commun. 2022 Apr 28;13:2325. doi: 10.1038/s41467-022-29967-1 (PMC9050736; doi:10.1038/s41467-022-29967-1)
Supplement: Supplementary file 2 — Description of Additional Supplementary Files [file 41467_2022_29967_MOESM2_ESM.pdf]

Description of Additional Supplementary Files

Supplementary Data 1

List of top 100 differentially expressed genes between low and high doses of CHIR.

Supplementary Data 2

Primer sequences for qPCR.

Supplementary Data 3

Statistics of scRNA-seq.

Supplementary Movie 1

Time-lapse imaging of three types of somitoids on day 6. Hours: Minutes. The snapshot of Separated paired somitoid is also shown in Fig. 1c. Time-lapse images were taken by an Opera Phenix HSC system.

Supplementary Movie 2

3D reconstruction of a day 7 somitoid showing the alternating patterns of *UNCX4.1* and *TBX18* expressions. The snapshot is also shown in Fig. 1g. Images were taken by a MuVi-SPIM Light-Sheet Microscope.

Supplementary Movie 3

Z-stacks of a day 7 somitoid showing cells between paired somites. Z-stack interval is 3 µm. The snapshot is also shown in Supplementary Fig. 7b. Images were taken by an LSM 980 Confocal microscope.

Supplementary Movie 4

Simultaneous monitoring of the HES7 reporter activity and somite formation from day 4 onward. The movie was stopped a few times to adjust the sample position. Bright field, HES7 reporter, and merged images are shown. Black arrows indicate the timings of new somite boundary formation. The snapshots are also shown in Fig. 3a,b. Time-lapse images were taken by an LV200 luminescent microscope.

Supplementary Movie 5

Time-lapse imaging of somitoids from day 6 onward without (W/O) or with different concentrations of Matrigel. Hours: Minutes. The snapshots of 5, 25, and 50% Matrigel are also shown in Supplementary Fig. 14a. Time-lapse images were taken by an Opera Phenix HSC system.
