# Supplementary material for: Periodic formation of epithelial somites from human pluripotent stem cells
Source: Nat Commun. 2022 Apr 28;13:2325. doi: 10.1038/s41467-022-29967-1 (PMC9050736; doi:10.1038/s41467-022-29967-1)
Supplement: Supplementary file 11 — Reporting Summary [file 41467_2022_29967_MOESM11_ESM.pdf]

Corresponding author(s): Marina Sanaki-Matsumiya, Miki Ebisuya

Last updated by author(s): Mar 30, 2022

Reporting Summary

Nature Portfolio wishes to improve the reproducibility of the work that we publish. This form provides structure for consistency and transparency in reporting. For further information on Nature Portfolio policies, see our Editorial Policies and the Editorial Policy Checklist.

Statistics

For all statistical analyses, confirm that the following items are present in the figure legend, table legend, main text, or Methods section.

n/a	Confirmed	
☐	☒	The exact sample size (*n*) for each experimental group/condition, given as a discrete number and unit of measurement
☐	☒	A statement on whether measurements were taken from distinct samples or whether the same sample was measured repeatedly
☐	☒	The statistical test(s) used AND whether they are one- or two-sided<br>*Only common tests should be described solely by name; describe more complex techniques in the Methods section.*
☒	☐	A description of all covariates tested
☒	☐	A description of any assumptions or corrections, such as tests of normality and adjustment for multiple comparisons
☐	☒	A full description of the statistical parameters including central tendency (e.g. means) or other basic estimates (e.g. regression coefficient) AND variation (e.g. standard deviation) or associated estimates of uncertainty (e.g. confidence intervals)
☐	☒	For null hypothesis testing, the test statistic (e.g. *F*, *t*, *r*) with confidence intervals, effect sizes, degrees of freedom and *P* value noted<br>*Give P values as exact values whenever suitable.*
☒	☐	For Bayesian analysis, information on the choice of priors and Markov chain Monte Carlo settings
☒	☐	For hierarchical and complex designs, identification of the appropriate level for tests and full reporting of outcomes
☒	☐	Estimates of effect sizes (e.g. Cohen's *d*, Pearson's *r*), indicating how they were calculated

Our web collection on statistics for biologists contains articles on many of the points above.

Software and code

Policy information about availability of computer code

Data collection	The following softwares were used: Olympus FV3000 Fluoview RS software for data collection by FV3000 confocal microscopy, Harmony software v4.9 for data collection by Opera Phenix HSC, Luxendo processor software v3.0 for data collection by MuViSpim CS, cellSens Dimension software for data collection by LV200, Kronos control software v2.3 for data collection by for data collection by Atto, and ZEN for data collection by Airyscan2 LSM980.
Data analysis	Image processing and analysis were perfomed using Fiji(v. 1.53f51), python (v. 3.7, v. 3.8), and R (v. 4.0.3). Subsequent analysis was performed on custom python and R scripts, MOrgAna, and pyBOAT.<br>scRNA-seq analysis was performed using CellRanger (v. 6.1.1), Python (v. 3.8.5), and R (v. 4.1.0, v. 4.1.2). We used custom python and R scripts with R package: Seurat (v.4.0.5), Scrublet (v.0.2.3), deMULTIplex (v.1.0.2), scran (v.1.22.1), scuttle (v.1.4.0), scater (v.1.22.0), batchelor (v.1.10.0), bluster (v. 1.4.0), Velocyto (v.0.17.17), scVelo (v.0.2.4), clusterProfiler (v.4.2.2).<br>Custom codes were deposited into GitHub [github.com/Nikoula86/2022_Somitoids_Analysis].

For manuscripts utilizing custom algorithms or software that are central to the research but not yet described in published literature, software must be made available to editors and reviewers. We strongly encourage code deposition in a community repository (e.g. GitHub). See the Nature Portfolio guidelines for submitting code & software for further information.

Data

Policy information about availability of data

All manuscripts must include a data availability statement. This statement should provide the following information, where applicable:

- Accession codes, unique identifiers, or web links for publicly available datasets
- A description of any restrictions on data availability
- For clinical datasets or third party data, please ensure that the statement adheres to our policy

The scRNA-seq data are available at www.ebi.ac.uk/arrayexpress/experiments/E-MTAB-11334. The remaining data are available within the Article, Supplementary Information, or Source Data file.

Field-specific reporting

Please select the one below that is the best fit for your research. If you are not sure, read the appropriate sections before making your selection.

[x] Life sciences [ ] Behavioural & social sciences [ ] Ecological, evolutionary & environmental sciences

For a reference copy of the document with all sections, see nature.com/documents/nr-reporting-summary-flat.pdf

Life sciences study design

All studies must disclose on these points even when the disclosure is negative.

Sample size	No statistical method was used to predetermine the sample size, and it was chosen by common standards in the field. The actual sample size (N) is given in the figure legends. Minimum three samples were used, and all experiments were independently repeated at least three times except for Supplementary Fig. 11a (Negative control, two times) and scRNA-seq (no replicate).
Data exclusions	If cells did not aggregate on day 1 of the somitoid protocol due to cell culture conditions, the experiment was discontinued.
Replication	All attempts of replication have been successful. The variability was reported in the graphs and figure legends.
Randomization	Somitoids were created in the same condition within the same 96 well plate, and each well was randomly assigned for analysis.
Blinding	The investigators who ran sequencing for scRNA-seq analyses were not aware of the sample groups. Blinding was not used for most analyses since the measurements and classification of somitoids were either semi-automated or done with a clear definition.

Reporting for specific materials, systems and methods

We require information from authors about some types of materials, experimental systems and methods used in many studies. Here, indicate whether each material, system or method listed is relevant to your study. If you are not sure if a list item applies to your research, read the appropriate section before selecting a response.

Materials & experimental systems

n/a	Involved in the study	
[ ]	[x]	Antibodies
[ ]	[x]	Eukaryotic cell lines
[x]	[ ]	Palaeontology and archaeology
[x]	[ ]	Animals and other organisms
[x]	[ ]	Human research participants
[x]	[ ]	Clinical data
[x]	[ ]	Dual use research of concern

Methods

n/a	Involved in the study	
[x]	[ ]	ChIP-seq
[x]	[ ]	Flow cytometry
[x]	[ ]	MRI-based neuroimaging

Antibodies

Antibodies used

All antibodies used in this study are commercially available antibodies.

Primary antibody
anti-ZO-1 antibody, 1/300 dilution, Invitrogen, 61-7300
anti-TBX6 antibody, 1/300 dilution, abcam, ab38883
anti-SOX2 antibody, 1/300 dilution, R&D, MAB2018, Clone # 245610
anti-BRACHYURY antibody, 1/300 dilution, R&D, AF2085
anti-SOX1 antibody, 1/300 dilution, R&D, AF3369

anti-PAX6 antibody, 1/300 dilution, abcam, ab195045

Secondary antibodies
Alexa Fluor 488 Phalloidin, 1/300 dilution, Invitrogen, A12379
Alexa Fluor 594 Goat anti-Rabbit IgG (H+L), 1/500 dilution, Invitrogen, A-11037
Alexa Fluor 647 Donkey anti-Goat IgG (H+L), 1/500 dilution, Invitrogen, A32849
Alexa Fluor 647 Goat anti-Mouse IgG (H+L), 1/500 dilution, Invitrogen, A-21236
Alexa Fluor 488 Goat anti-Mouse IgG (H+L), 1/500 dilution, Invitrogen, A-11029
DAPI, 1/1000 dilution, Invitrogen, 62247

Validation

ZO-1, Invitrogen, # 61-7300 (https://www.thermofisher.com/antibody/product/ZO-1-Antibody-Polyclonal/61-7300)
TBX6, abcam, ab38883 (https://www.abcam.com/tbx6-antibody-ab38883.html)
SOX2, R&D, MAB2018 (https://www.rndsystems.com/products/human-mouse-rat-sox2-antibody-245610_mab2018)
BRACHYURY, R&D, AF2085 (https://www.rndsystems.com/products/human-mouse-brachyury-antibody_af2085)
SOX1, R&D, AF3369 (https://www.rndsystems.com/products/human-mouse-rat-sox1-antibody_af3369)
PAX6, abcam, ab195045 (https://www.abcam.com/pax6-antibody-epr15858-ab195045.html)

Alexa Fluor 594 Goat anti-Rabbit IgG (H+L), Invitrogen, A-11037 (https://www.thermofisher.com/antibody/product/Goat-anti-Rabbit-IgG-H-L-Highly-Cross-Adsorbed-Secondary-Antibody-Polyclonal/A-11037)
Alexa Fluor 647 Donkey anti-Goat IgG (H+L), Invitrogen, A32849 (https://www.fishersci.com/shop/products/igg-h-l-highly-cross-adsorbed-donkey-anti-goat-alexa-fluor-plus-647-polyclonal-secondary-antibody-invitrogen/PIA32849)
Alexa Fluor 647 Goat anti-Mouse IgG (H+L), Invitrogen, A-21236 (https://www.thermofisher.com/antibody/product/Goat-anti-Mouse-IgG-H-L-Highly-Cross-Adsorbed-Secondary-Antibody-Polyclonal/A-21236)
Alexa Fluor 488 Goat anti-Mouse IgG (H+L), Invitrogen, A-11029 (https://www.thermofisher.com/antibody/product/Goat-anti-Mouse-IgG-H-L-Highly-Cross-Adsorbed-Secondary-Antibody-Polyclonal/A-11029)
Alexa Fluor 488 Phalloidin, Invitrogen, A12379 (https://www.thermofisher.com/order/catalog/product/A12379#/A12379)

Eukaryotic cell lines

Policy information about cell lines

Cell line source(s)	Human iPS cell line (201B7, #HPS0063) was originally described in Takahashi et al., Cell, 131 (2007).<br>The cell line was provided by the RIKEN BRC through the National BioResource Project of the MEXT/AMED, Japan.
Authentication	The cell lines was not authenticated.
Mycoplasma contamination	Cells were regularly tested and reported negative for mycoplasma contamination.
Commonly misidentified lines<br>(See ICLAC register)	NA
